# The pediatric leukemia oncoprotein NUP98-KDM5A induces genomic instability that may facilitate malignant transformation

**DOI:** 10.1038/s41419-023-05870-5

**Published:** 2023-06-10

**Authors:** Joan Domingo-Reinés, Rosa Montes, Adrián Garcia-Moreno, Amador Gallardo, Jose Manuel Sanchez-Manas, Iván Ellson, Mar Lamolda, Chiara Calabro, Jose Antonio López-Escamez, Purificación Catalina, Pedro Carmona-Sáez, Pedro J. Real, David Landeira, Verónica Ramos-Mejia

**Affiliations:** 1 GENYO, Centre for Genomics and Oncological Research Pfizer - University of Granada - Andalusian Regional Government, PTS, 18016 Granada, Spain; 2 Department of Cell Biology, Faculty of Sciences, University of Granada, 18071 Granada, Spain; 3 Department of Biochemistry and Molecular Biology II, Faculty of Pharmacy, University of Granada, 18071 Granada, Spain; 4 Instituto de Investigación Biosanitaria ibs.GRANADA, Granada, Spain; 5 Department of Biochemistry and Molecular Biology I, Faculty of Sciences, University of Granada, 18071 Granada, Spain; 6 Sensorineural Pathology Programme, Centro de Investigación Biomédica en Red en Enfermedades Raras, CIBERER, Madrid, Spain; 7 Meniere’s Disease Neuroscience Research Program, Faculty of Medicine & Health, School of Medical Sciences, The Kolling Institute, University of Sydney, Sydney, NSW Australia; 8 Andalusian Public Health System Biobank, Coordinating Node, Av. del Conocimiento, S/N, 18016 Granada, Spain; 9 Department of Statistics, University of Granada, 18071 Granada, Spain; 10 Excellence Research Unit “Modeling Nature” (MNat), University of Granada, 18016 Granada, Spain

**Keywords:** Cancer models, Acute myeloid leukaemia

## Abstract

Pediatric Acute Myeloid Leukemia (AML) is a rare and heterogeneous disease characterized by a high prevalence of gene fusions as driver mutations. Despite the improvement of survival in the last years, about 50% of patients still experience a relapse. It is not possible to improve prognosis only with further intensification of chemotherapy, as come with a severe cost to the health of patients, often resulting in treatment-related death or long-term sequels. To design more effective and less toxic therapies we need a better understanding of pediatric AML biology. The NUP98-KDM5A chimeric protein is exclusively found in a particular subgroup of young pediatric AML patients with complex karyotypes and poor prognosis. In this study, we investigated the impact of NUP98-KDM5A expression on cellular processes in human Pluripotent Stem Cell models and a patient-derived cell line. We found that NUP98-KDM5A generates genomic instability through two complementary mechanisms that involve accumulation of DNA damage and direct interference of RAE1 activity during mitosis. Overall, our data support that NUP98-KDM5A promotes genomic instability and likely contributes to malignant transformation.

## Introduction

Acute myeloid leukemia (AML) is a rare and heterogeneous disease that contributes to 20% of all pediatric leukemia, but is becoming the leading cause of death of children with leukemia [1]. Although their outcome have improved in the last years [2, 3], the cure rates remain unsatisfactory low for some AML subtypes and current therapies often result in treatment-related deaths as well as long term secondary effects [1, 4, 5]. Therefore, we need to better understand pediatric leukemia biology to develop more effective and less toxic targeted therapies and improve the outcome in pediatric AML [4].

The dominant hypothesis suggests that most pediatric leukemias originate by the acquisition of driving genetic alterations before birth [6, 7]. Chromosomal translocations are very often the initiating event and it is suggested that the resulting fusion oncoprotein might contribute to the acquisition of the additional molecular alterations needed to develop leukemia [6, 7]. Pediatric AML patients carrying NUP98 chimeric gene rearrangements represent a singular subgroup with poor prognosis, high rates of induction failure and chemotherapy resistance [1, 5, 8, 9]. In particular, around 2% of all pediatric AML patients harbor the chromosomal translocation t(11;12)(p15;p13), that gives rise to the chimeric fusion of the nucleoporin 98 (*NUP98*) and the *KDM5A* lysine demethylase, resulting in NUP98-KDM5A fusion protein [10, 11]. Patients carrying NUP98-KDM5A fusion present an unfavorable prognosis and high relapse rates [1, 11, 12]. NUP98-KDM5A is most frequently found in acute megakaryoblastic leukemia but is also observed in all AML subtypes, associated with complex karyotypes and cooperating mutations in *KRAS* and *RB1* [1, 10–14]. NUP98-KDM5A is found in very young pediatric patients with a median age of 3 years old[1, 11, 12], suggesting that the t(11;12) translocation occurs *in utero*.

Human Pluripotent Stem Cells (hPSC) provide unlimited numbers of cells to investigate the physiopathology of human diseases [15]. Since hPSC and their derivatives resemble embryonic/fetal stages of human development [16], they constitute an ontologically ideal model for studying early onset diseases.

Here, we generated human Pluripotent Stem Cell models constitutively expressing NUP98-KDM5A. We found that this fusion protein leads to an increase in production of DNA double-strand breaks (DSBs), aberrant mitosis and chromosome missegregation. Proteomics analysis showed that NUP98-KDM5A directly interferes with RAE1 activity during mitosis. Additionally, in the patient-derived cell line CHRF-288-11, which harbors the NUP98-KDM5A fusion, we found increased levels of DSBs and corroborated the direct interaction of NUP98-KDM5A with RAE1 during mitosis. These results indicate that NUP98-KDM5A induces genomic instability that may facilitate malignant transformation.

## Results

### Generation of NUP98-KDM5A-expressing hPSCs

**Fig. 1:**
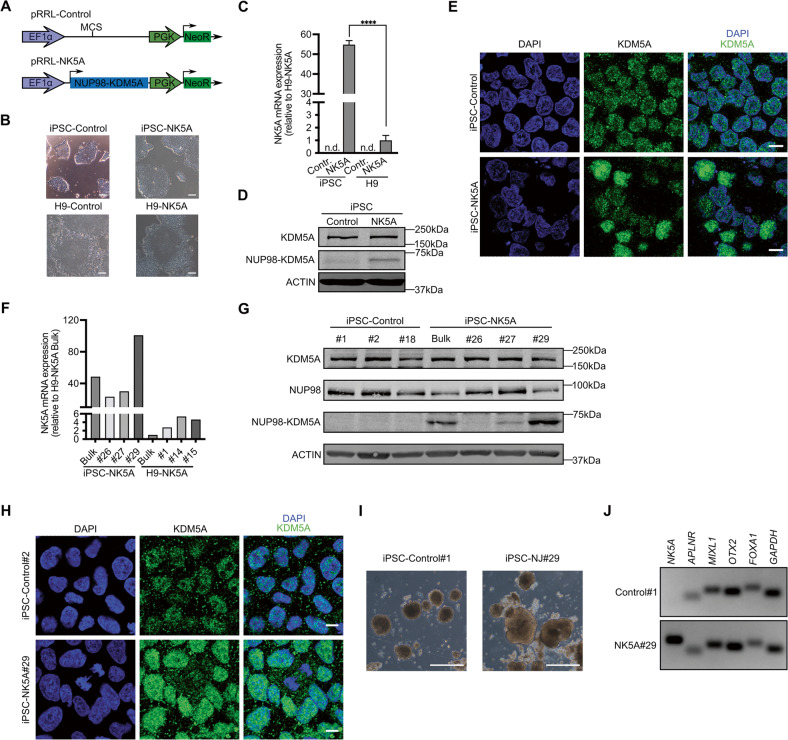
Generation of iPSC and H9 cell lines expressing the fusion protein NUP98-KDM5A. **A** Schematic representation of the lentiviral vectors used. MCS: Multi-cloning site. EF1α and PGK: promoters. **B** Representative images of hPSC transduced with the lentiviral vectors. Scale bar = 100 μm. **C** Quantitative real time polymerase chain reaction (qRT-PCR) analysis showing the expression of the fusion gene in H9 and iPSC (Control and NK5A), *n* = 3. **D** Western blot analysis detecting the endogenous KDM5A protein and NUP98-KDM5A fusion protein in the iPSC-control and iPSC-NK5A lines. Actin is used as a loading control. Molecular weights: KDM5A (196 kDa), NUP98-KDM5A (70 kDa) and ACTIN (42 kDa). **E** Immunofluorescence detecting endogenous KDM5A protein and NUP98-KDM5A fusion protein in iPSC-control and iPSC-NK5A lines. Scale bar = 10 μm. **F** Relative expression of *NUP98-KDM5A* analyzed by qRT-PCR in iPSC-KDM5A and H9-KDM5A clones. **G** Western blot analysis detecting the endogenous KDM5A and NUP98 proteins and NUP98-KDM5A fusion protein in the iPSC clones. ACTIN is used as a loading control. **H** Immunofluorescence detecting endogenous KDM5A and NUP98-KDM5A in iPSC-C#1 and iPSC-NK5A#29. Scale bar = 10 μm. **I** Images of EBs of iPSC-C#1 and iPSC-NK5A#29 at day 9 of differentiation. Scale bar = 500 μm. **J** Agarose gel of PCR amplified genes: *NK5A* (*NUP98-KDM5A*) 133 bp, *APLNR*, 62 bp and *MIXL1*, 106 bp (Mesoderm), *OTX2*, 96 bp (Ectoderm), *FOXA1*, 122 bp (Endoderm) and *GAPDH*, 87 bp (Housekeeping). Data in plots indicate mean ± SEM. ****p < 0.0001; Two-tailed unpaired Student’s *t*-student test applied (**C**).

We cloned the NUP98-KDM5A cDNA into a bicistronic vector harboring the neomycin resistance gene and transduced the H9 line and the iPSC line, with either the empty vector (control) or the NUP98-KDM5A-expressing vector (NK5A) (Fig. 1A). After neomycin selection, NK5A-expressing hPSCs display the typical morphology of pluripotent stem cells, growing in compact colonies with tightly packed cells (Fig. 1B). Transgenic hPSCs expressed the pluripotency transcription factors *POU5F1*, *NANOG* and *SOX2* (Supplementary Fig. 1A, B), the pluripotent markers SSEA-4 and TRA-1-60 (Supplementary Fig. 1C), and they were positive for alkaline phosphatase activity (Supplementary Fig. 1D, E).

By quantitative PCR (qPCR) we determined that in the iPSC-NK5A line the expression of the *NUP98-KDM5A* RNA was nearly 50-fold higher that in the H9-NK5A line (Fig. 1C). Expression of *NK5A* did not affect the expression levels of the endogenous *NUP98* and *KDM5A* genes in both hPSC-NK5A lines (Supplementary Fig. 1F, G).

We confirmed NUP98-KDM5A protein expression by Western Blot (Fig. 1D and supplementary material) and by immunofluorescence (Fig. 1E and Supplementary Fig. 1H), using an antibody directed against KDM5A that also recognized the fusion protein. By Western Blot we detected the endogenous expression of KDM5A protein (196 kDa) in iPSC-NK5A and iPSC control lines, but only in iPSC-NK5A line we identified a band of 70 kDa, corresponding to the NUP98-KDM5A fusion protein according to its expected molecular weight (Fig. 1D and supplementary material). Immunofluorescence analyses revealed a nuclear punctate staining in hPSC-controls and hPSC-NK5A lines, corresponding to the endogenous KDM5A protein (Fig. 1E and Supplementary Fig. 1H). Bulk cultures of both NUP98-KDM5A-expressing hPSCs displayed evident heterogeneity of fluorescence intensity, indicating that only a proportion of the cells were expressing the NUP98-KDM5A fusion protein (Fig. 1E and Supplementary Fig. 1H).

To establish hPSC lines with stable and homogeneous NUP98-KDM5A expression, we generated clones by limiting dilution and obtained clonal lines that express different levels of *NUP98-KDM5A* mRNA expression (Supplementary Fig. 1I). We selected six clones based on their distinct *NUP98-KDM5A* mRNA expression levels for further characterization: clones H9-NK5A- #1, #14 and #15 with low expression, and clones iPSC-NK5A #26, #27 and #29 with higher expression levels (Fig. 1F). The level of expression of NUP98-KDM5A protein mirrored the amount of mRNA at individual iPSC-KDM5A clonal lines (Fig. 1F, G, Supplementary Fig. 1J, K and supplementary material). NUP98-KDM5A-expressing clones displayed homogeneous expression of the fusion protein (Fig. 1H), maintained the pluripotent morphology, retained the expression of the pluripotency markers *POU5F1*, *NANOG* and *SOX2*, the antigens TRA-1-60 and SSEA-4, and the alkaline phosphatase activity (data not shown). Functionally, the hPSC-NK5A clones differentiated within embryoid bodies into progeny representing endoderm (*FOXA*+), mesoderm (*APLNR*+ and *MIXL*+), and ectoderm (*OTX*+) lineages (Fig. 1I, J). These results indicate that expression of NUP98-KDM5A fusion protein is compatible with the maintenance of the undifferentiated state of hPSCs.

### Expression of NUP98-KDM5A produces stress vulnerability, accumulation of cells in G2/M phases and a higher apoptosis rate

**Fig. 2:**
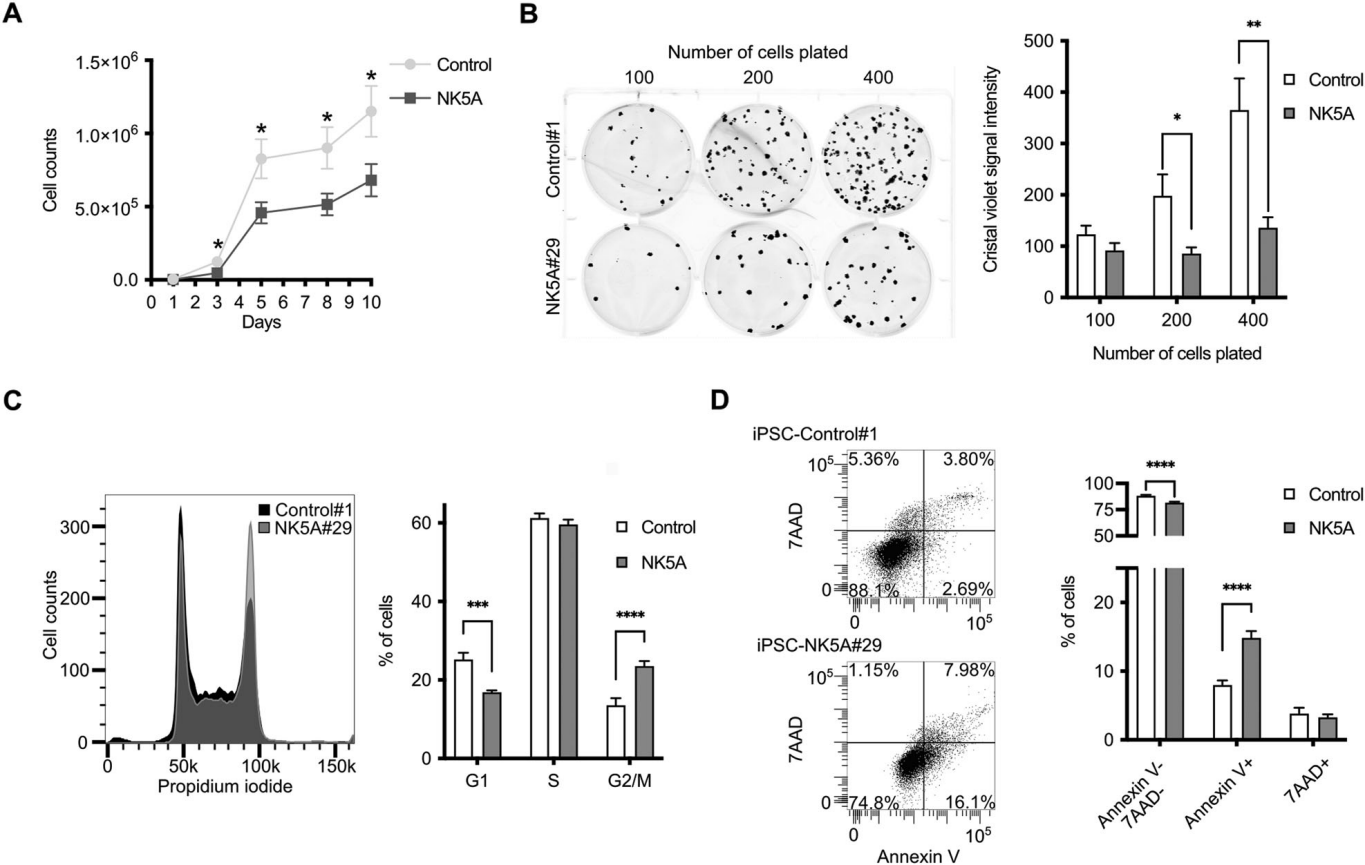
High expression of NUP98-KDM5A affects key biological functions in iPSC. **A** Growth curve of the iPSC-control and NK5A clones at day 3, 5, 8 and 10, *n* = 9. **B** Left, Representative images of colony forming unit assay at day 10. Right, crystal violet quantification of the iPSC-control and NK5A clones using 100, 200 and 400 initial cells at day 10, *n* = 9. **C** Left, representative images of cell cycle profile of iPSC-C#1 and NK5A#29. Right, graph showing the percentage of cells in each phase of the cell cycle in iPSC-control and NK5A clones, *n* = 9. **D** Left, representative flow cytometry dot plots of apoptosis quantification. Right, graph showing the percentage of viable, apoptotic, and necrotic populations in iPSC-control and NK5A clones, *n* = 9. Data are represented as mean ± SEM. *p < 0.05, **p < 0.01, ***p < 0.001, ****p < 0.0001; Two-tailed paired Student’s t-student test applied (**A**), Two-way ANOVA test applied in (**B**, **C**, **D**).

We observed that iPSC-NK5A clones had a slower growth rate than control clones (Fig. 2A). In contrast, we did not notice any growth difference between H9-NK5A and H9 control lines. We assessed cell survival by plating at low density (100, 200 and 400 cells) single cells from hPSC-NK5A and control hPSCs (Fig. 2B). Colony Forming Unit (CFU) assay showed a significant reduction in the resulting colonies of the iPSC-NK5A lines as compared with controls after platting 200 and 400 cells (Fig. 2B). In contrast, lower expression of NUP98-KDM5A did not affect the number of CFUs (H9-NK5A *vs* H9 control, Supplementary Fig. 2A). These results suggest that the expression level of NUP98-KDM5A above a certain threshold diminishes the ability of cells to cope with cell culture stress.

Next, we analyzed the distribution of cell cycle phases in hPSC-NK5A and control clones using flow cytometry. We did not observe differences in cell cycle profiles between H9-NK5A and H9-control (Supplementary Fig. 2B), but we detected significant differences in iPSC-NK5A in comparison with iPSC-control (Fig. 2C). In iPSC-NK5A clones the percentages of cells in the G1 phase were significantly reduced (16.8 ± 0.52% *vs* 25.2 ± 1.72%), in the G2/M phase were significantly increased (23.5 ± 1.24 *vs* 13.5 ± 1.79%), and there were no changes in S phase (59.5 ± 1.33% *vs* 61.2 ± 1.20%), as compared with control lines (Fig. 2C). In addition, Annexin V staining showed significantly higher percentage of apoptotic cells in iPSC-NK5A clones than in controls, 15 ± 0.96% *vs* 8 ± 0.69%, respectively (Fig. 2D).

Overall, hPSC expressing higher levels of NUP98-KDM5A displayed increased stress sensitivity, an accumulation of cells in the G2/M phases and higher apoptosis rates.

### NUP98-KDM5A leads to transcriptional up-regulation of HIF1A target genes

**Fig. 3:**
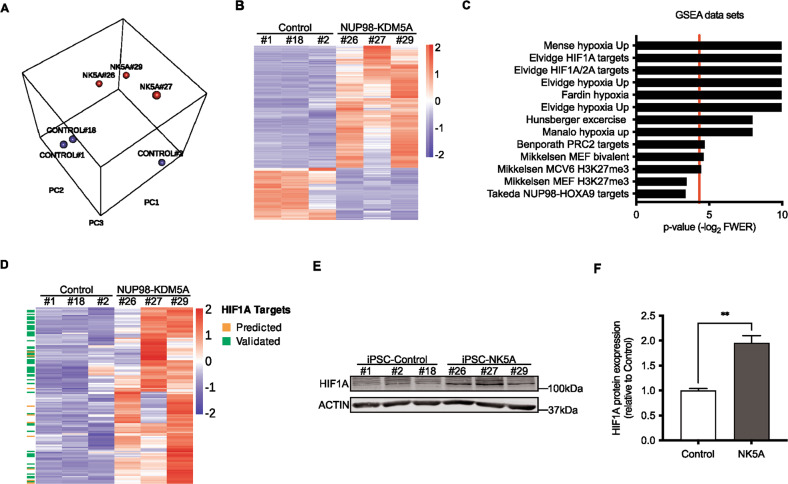
Transcriptomic analysis of NUP98-KDM5A expressing-iPSC. **A** Principal Component Analysis for RNA-seq data in iPSC-control and iPSC-NK5A clones. **B** Hierarchical clustering heat map of the differentially expressed genes (fold change log_2_ > 1 or <−1 and adjusted *p* value < 0.05) between iPSC-control and iPSC-NK5A clones. **C** Top 13 ranked gene sets in the GSEA of genes up-regulated in NK5A expressing iPSCs compared with control samples. **D** Hierarchical clustering heat map of TOP8 GSEA hypoxia-related gene sets. Direct targets of HIF1A from Harmonizome data base are highlighted: Validated (green) and Predicted (orange). **E** Western blot analysis detecting HIF1A in iPSC-control and iPSC-NK5A clones. ACTIN is used as a loading control. **F** Quantification of the levels of HIF1A protein in iPSC-control and NK5A clones, *n* = 3. Red line in (**C**) is *p* values < 0.05. **p < 0.01; Two-tailed unpaired Student’s *t*-student test applied in (**F**).

To evaluate the molecular consequences of NUP98-KDM5A expression, we compared the whole transcriptome of iPSC-NK5A and control lines using RNA-seq. Principal component analysis showed a clear cluster separation of the NK5A-iPSCs and control clones (Fig. 3A). A total of 652 genes were differentially expressed in iPSC-NK5A cells compared to controls (adjusted *p* value < 0.05 and absolute log2 fold-change >1). Differentially expressed genes were unequally distributed, as 70% were upregulated (475 genes) and 30% down-regulated (195 genes) (Fig. 3B and Supplementary Fig. 3A), in accordance with the reported function of NUP98-KDM5A in preventing gene silencing [17] Gene set enrichment analysis showed 55 enriched gene sets (adjusted *p* value < 0.05) (Supplementary Table 1) that included targets genes of NUP98-HOXA9 fusion in hematopoietic progenitors at the initial stages of leukemogenesis [18] (Fig. 3C and Supplementary Fig. 3B). Among the gene sets that were significantly enriched (adjusted *p* value < 0.05), two were related to bivalent chromatin genes and Polycomb Repressive Complex 2 (PRC2) targets (Fig. 3C), which is consistent with the role of KDM5A regulating transcription of bivalent promoters [19–21].

Importantly, seven out of the eleven significantly enriched gene sets were directly associated with hypoxia *(*Fig. 3C and Supplementary Fig. 3B). Given that hypoxia is largely regulated by the Hypoxia-inducible factor (HIF) at the transcriptional level [22, 23], we investigated the implication of HIF on gene expression changes by analyzing the leading edge genes of the top eight significantly up-regulated gene sets using ChEA-ChIP-X Enrichment Analysis [24] and TRANSFAC [25] databases. We found that 43% of the deregulated genes (61/140) were direct targets of HIF1A (Fig. 3D).

The over-representation of genes regulated by HIF1A in the transcriptomic analysis led us to investigate the HIF1A protein by Western blot of whole-cell extracts from iPSC-NK5A and control clones. Under normoxic conditions, HIF1A protein is degraded and stabilized by hypoxia; [22] accordingly HIF1A was almost undetectable in controls (Fig. 3E). By contrast, in iPSC-NK5A clones HIF1A protein expression was significantly increased (Fig. 3E, F), indicating that HIF1A is stabilized in these lines and supporting that NUP98-KDM5A expression induces a hypoxic gene signature mediated by HIF1A.

### Increased DNA damage in NUP98-KDM5A-expressing iPSC

**Fig. 4:**
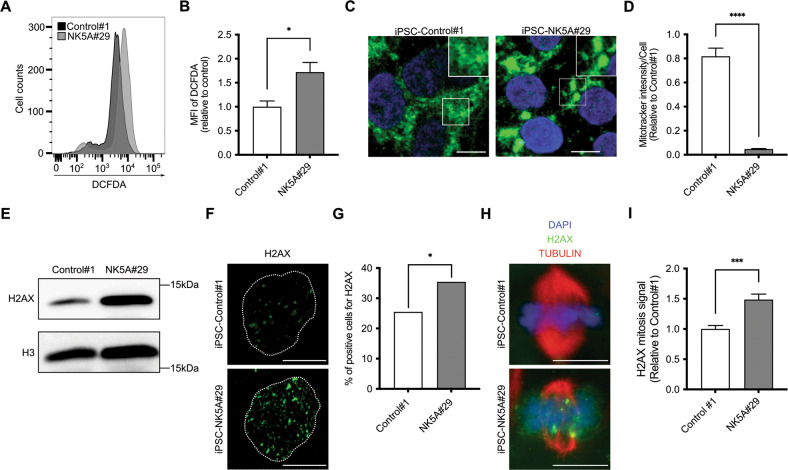
NUP98-KDM5A increases ROS production and DNA damage. **A** Reactive oxygen species (ROS) analysis detecting DCFDA in the iPSC-C#1 and NK5A#29 lines. **B** Median fluorescence intensity (MFI) of the DCFDA in iPSC-C#1 and NK5A#29, *n* = 3. **C** Representative immunofluorescence visualizing the mitochondria using Mitotracker 633 in iPSC-Control#1 and NK5A#29. DAPI (blue), Mitotracker 633 (green). Scale bar = 10 μm. **D** Mean signal of Mitotracker 633. Control#1, *n* = 137 nuclei, NK5A#29, *n* = 121 nuclei. **E** Western blot analysis detecting H2AX in iPSC-C#1 and NK5A#29. H3 is used as a loading control. **F** Representative image showing one nucleus of the immunofluorescence detecting H2AX in iPSC-C#1 and NK5A#29. The nucleus is represented as a discontinuous line. Scale bar = 10 μm. **G** Percentage of positive cells for H2AX. Control#1, *n* = 300 nuclei, NK5A#29, *n* = 300 nuclei. **H** Representative image showing one metaphase of the immunofluorescence detecting H2AX and α-Tubulin in mitosis of iPSC-C#1 and NK5A#29. Scale bar = 10 μm. **I** Mean intensity signal of H2AX in mitotic cells. Control#1, *n* = 14 mitosis, NK5A#29, *n* = 22 mitosis. Data in plots indicate mean ± SEM (**B**, **D**, **I**) and % (**G**). *p < 0.05, ***p < 0.001, ****p < 0.0001; Two-tailed unpaired Student’s t-student test applied (**B**), Mann-Whitney test applied (**D**, **I**) and Chi-square test applied (**G**).

Reactive oxygen species (ROS) can stabilize HIF1A under normoxic conditions [26], therefore we determined ROS production by flow cytometry. We found that iPSC-NK5A cells significantly increased ROS production as compared with controls, suggesting that ROS over-production stabilized HIF1A (Fig. 4A, B). Since mitochondria are the major source of ROS that are essential for HIF1 stabilization in nonhypoxic conditions [27], we stained mitochondria with MitoTracker to analyze their morphology and mass. As expected, control cells showed prominent and mainly filamentous networked mitochondria (Fig. 4C, left panel). In contrast, iPSC-NK5A displayed few fuzzy, fragmented and clustered mitochondria (Fig. 4C, right panel). In addition, signal quantification revealed a significant decrease in total mitochondrial mass in NUP98-KDM5A-expressing iPSC as compared with control (Fig. 4D).

Considering that an excess of ROS is harmful for cells and causes DNA damage by oxidative stress [28], we assessed the expression of phosphorylated histone H2AX (γ-H2AX), which detect the formation of DNA double strand breaks (DSB) and is implicated in activation of DNA damage response (DDR) [28]. We observed increased levels of γ-H2AX in NUP98-KDM5A-expressing iPSC by Western Blot (Fig. 4E and supplementary material). We also found significantly higher number of γ-H2AX^+^ cells in iPSC-NK5A as compared with iPSC-control (Fig. 4F–I) by immunofluorescence. The increased levels of γ-H2AX expression were significantly relevant during mitosis, indicating the activation of the DDR mechanism in NUP98-KDM5A-expressing iPSC during cell division (Fig. 4H, I).

These results indicate that NUP98-KDM5A expression in iPSCs increases ROS production and affects mitochondria homeostasis, leading to increased levels of DNA damage.

### Interaction of NUP98-KDM5A and RAE1 leads to mitotic defects

**Fig. 5:**
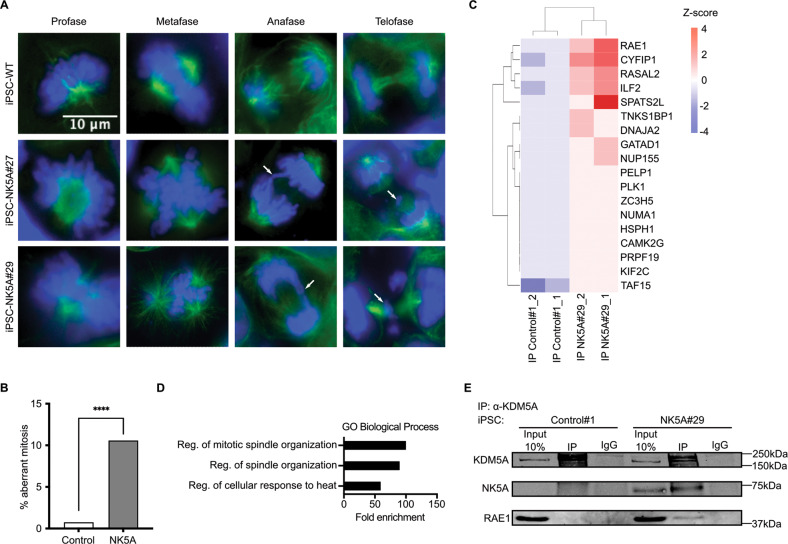
NUP98-JARI1A produces aberrant mitosis through RAE1 interaction. **A** Immunofluorescence detecting α-tubulin (green) and DNA (blue) of iPSC-WT, iPSC-NK5A#27 and NK5A#29. Representative images showing each mitosis phase. **B** Quantification of the aberrant mitosis in the immunofluorescence of α-tubulin comparing the control group (iPSC-WT and C#2) against the fusion protein expressing group (iPSC-NK5A#27 and NK5A#29), *n* = 200 mitosis per cell line. **C** Hierarchical heatmap of z-score values of the NUP98-KDM5A candidate interacting proteins. **D** Gene ontology (GO) of biological process of NUP98-KDM5A interactors. Results are displayed for FDR *p* < 0.05. **E** Western blot analysis with anti-KDM5A and anti-RAE1 antibodies of the pulldowns against KDM5A of nocodazole-arrested iPSC-control#1 and NK5A#29. ****p < 0.0001; Chi-square test applied in (**B**).

As shown above, NUP98-KDM5A expression led to DDR during mitosis and an accumulation of cells in G2/M phases, which could reflect abnormalities during mitotic progression [29, 30]. To investigate whether NUP98-KDM5A expression affects the progression of the cells through mitosis, we performed immunofluorescence staining against α-tubulin to analyze mitotic spindle formation and DAPI staining to observe the segregation of chromosomes. The iPSC-controls mainly displayed normal mitosis (Fig. 5A, top row). In contrast, the iPSC-NK5A clones displayed multiple mitotic errors including multipole formation, chromosome bridges and lagging chromosomes (Fig. 5A, middle and bottom rows). In iPSC-NK5A clones up to 10% of the observed mitosis were abnormal (Fig. 5B). It has been shown that some NUP98 fusion proteins can produce aberrant mitosis due to a direct interaction with CDC20, the coactivator of the anaphase-promoting complex (APC) during early mitosis, and interfere with the function of the APC/CCDC20 complex [31, 32]. To assess whether NUP98-KDM5A could be interacting with CDC20 during mitosis we performed double-immunofluorescence staining, but we did not find co-localization of NUP98-KDM5A with CDC20 (Supplementary Fig. 4).

We decided to carry out proteomic analysis to identify the NUP98-KDM5A interacting protein(s) that might account for the aberrant mitotic phenotype. We conducted two independent experiments of liquid chromatography tandem mass spectrometry on mitotic extracts of iPSC-NK5A#29 and control#1 clones, after immunoprecipitation (IP) with the KDM5A antibody. Since the anti KDM5A antibody recognizes the endogenous KDM5A and the NUP98-KDM5A, to identify specific interactions of the fusion protein we excluded from the analysis all the proteins appearing in the IPs of iPSC-Control#1. We identified eighteen NUP98-KDM5A candidate interacting proteins and generated a hierarchical heatmap of z-score values (Fig. 5C). Analysis of the list of proteins using the Protein ANalysis THrough Evolutionary Relationships (PANTHER) tool [33] (FDR < 0.05 and raw p-value < 0.05) revealed that the first two enriched Gene Ontology Biological Process annotations implicated the mitotic spindle organization (Fig. 5D). Among all the NUP98-KDM5A binding partners, the first one was the RNA export 1 (RAE1) protein. RAE1 interacts with NUP98, and during mitosis NUP98-RAE1 form a complex with APC/CDH1 and inhibit securin degradation to prevent aneuploidy [34]. It has been shown that RAE1 can interact with NUP98 fusion proteins through the GLEBS domain of the N terminal of NUP98 that is conserved in all NUP98 fusion proteins [32, 35]. Therefore, to validate NUP98-KDM5A-RAE1 interaction during mitosis, we pulled down NUP98-KDM5A from mitotic extracts of iPSC-NK5A and control clones and analyzed by Western Blot using an anti-RAE1 antibody. As is shown in Fig. 5E, RAE1 was detected only in NUP98-KDM5A-expressing iPSC.

Overall, we found that NUP98-KDM5A interacts directly with RAE1 during mitosis, likely contributing to the failure in mitotic spindle formation and chromosome segregation.

### The patient-derived hematopoietic cell line exhibits DNA damage and interaction between NUP98-KDM5A and RAE1

**Fig. 6:**
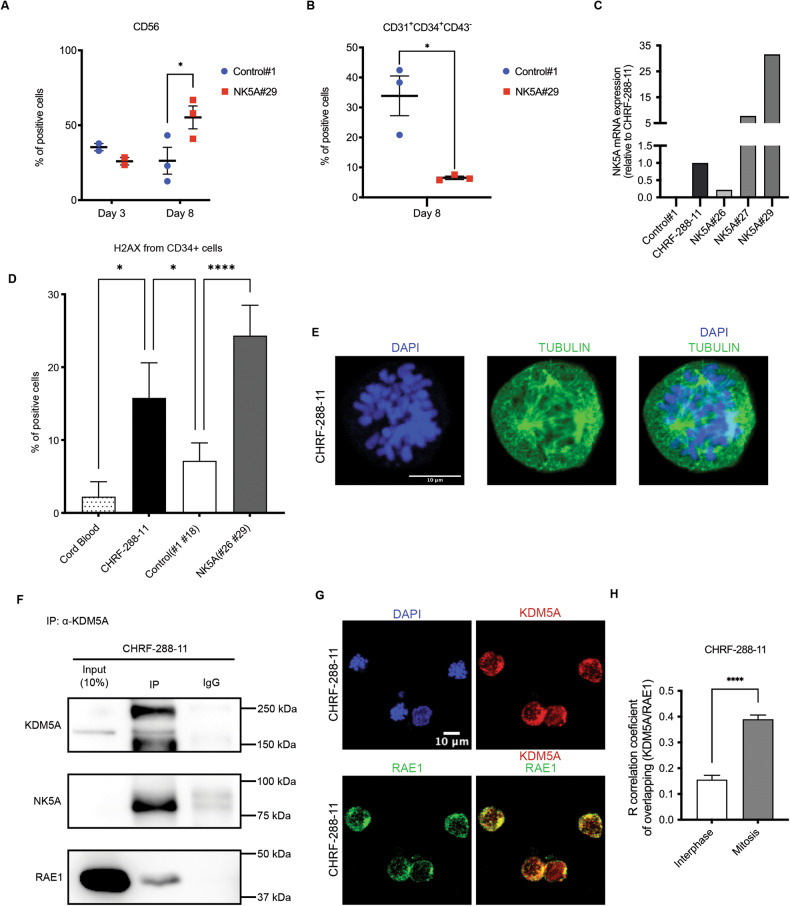
The CHRF-288-11 cell line presents DNA damage and interaction between NUP98-KDM5A and RAE1. Flow cytometry analysis of (**A**) mesoderm progenitors (CD56+) at day 3 and 8, and **B** hematoendothelial progenitors (CD31 + CD34 + CD43-) at day 8, during hematopoietic differentiation of NK5A#29 and C#1, *n* = 3. **C** Analysis of *NUP98-KDM5A* expression by qRT-PCR in CHRF-288-11, C#1 and iPSC-NK5A clones. **D** Flow cytometry analysis of H2AX expression in CD34+ cells produced by the iPSC-Control#1, #18 (Control, *n* = 6), iPSC-NK5A#26, NK5A#29 (NK5A, *n* = 6), the CHRF-288-11 cell line (*n* = 6) and Cord Blood-CD34+ cells (*n* = 2). **E** Representative image of the immunofluorescence detecting α-TUBULIN (green) and DNA (blue) of CHRF-288-11 cell line. **F** Western blot analysis with anti-KDM5A and anti-RAE1 antibodies of the pulldowns against KDM5A of nocodazole-arrested CHRF-288-11 cell line. **G** Representative image of the immunofluorescence detecting RAE1 (green), KDM5A (Red) and DNA (blue) of CHRF-288-11 cell line. **H** R correlation of overlapping between KDM5A and RAE1 in interphase or mitosis of CHRF-288-11 cells. *p < 0.05, ***p < 0.0005, ****p < 0.0001; Two-way ANOVA test applied in (**A** and **D**), Two-tailed unpaired Student’s *t*-student test applied in (**B** and **H**).

To analyze the effects of NUP98-KDM5A expression in a hematopoietic cellular context, we differentiated the NK5A#29 and Control#1 clones into hematopoietic progenitors. At day 8 of differentiation, in the NK5A#29 clone we observed the accumulation of CD56+ mesoderm progenitors but very few CD31 + CD34 + CD43- hematoendothelial progenitors that did not further differentiate (Fig. 6A, B). Given that NUP98-KDM5A expression in undifferentiated iPSC cells had a negative impact on hematopoietic differentiation, we decided to corroborate our findings in the CHRF-288-11 patient-derived cell line, which harbor de NUP98-KDM5A fusion [11]. By qPCR we found that *NUP98-KDM5A* expression level in the CHRF-288-11 line is within the range of expression of NK5A#26 and NK5A#27 clones, although is 30-fold higher in the NK5A#29 clone (Fig. 6C). We also evaluated the accumulation of DSBs in the CHRF-288-11 line, cord blood-CD34+ cells, and CD34+ cells produced by NK5A-iPSC and control clones. By flow cytometry we observed a significant increase of γ-H2AX+ cells in the NUP98-KDM5A expressing cells in comparison to CD34+ control cells (Fig. 6D). To evaluate whether the effects of NUP98-KDM5A in the mitotic process were reproducible in the CHRF-288-11 line, we analyzed the mitotic spindle formation by immunostaining against α-tubulin and verify the formation of multipoles (Fig. 6E).

Then, we pulled down NUP98-KDM5A from mitotic extracts of CHRF-288-11 cells, analyzed by Western Blot using the anti-RAE1 antibody and confirmed that NUP98-KDM5A interacts with RAE1 (Fig. 6F). To further corroborate the interaction between NUP98-KDM5A with RAE1, we analyzed their co-localization by immunofluorescence. With the anti-KDM5A we observed intense bright spots that likely correspond to NUP98-KDM5A fusion protein (Fig. 6G) and we also observed fluorescence co-localization with anti-RAE1 within these bright spots (Fig. 6G). We perform a co-localization analysis by evaluating the R coefficient of overlapping using ZEISS ZEN Black Microscopy Software, which revealed that the co-localization was significantly higher during mitosis (Fig. 6G).

These results confirm that patient-derived hematopoietic cells expressing NUP98-KDM5A exhibit accumulation of DNA damage, mitotic defects, and direct interaction of the fusion protein with RAE1 during mitosis.

### NUP98-KDM5A expression promotes chromosome instability

**Fig. 7:**
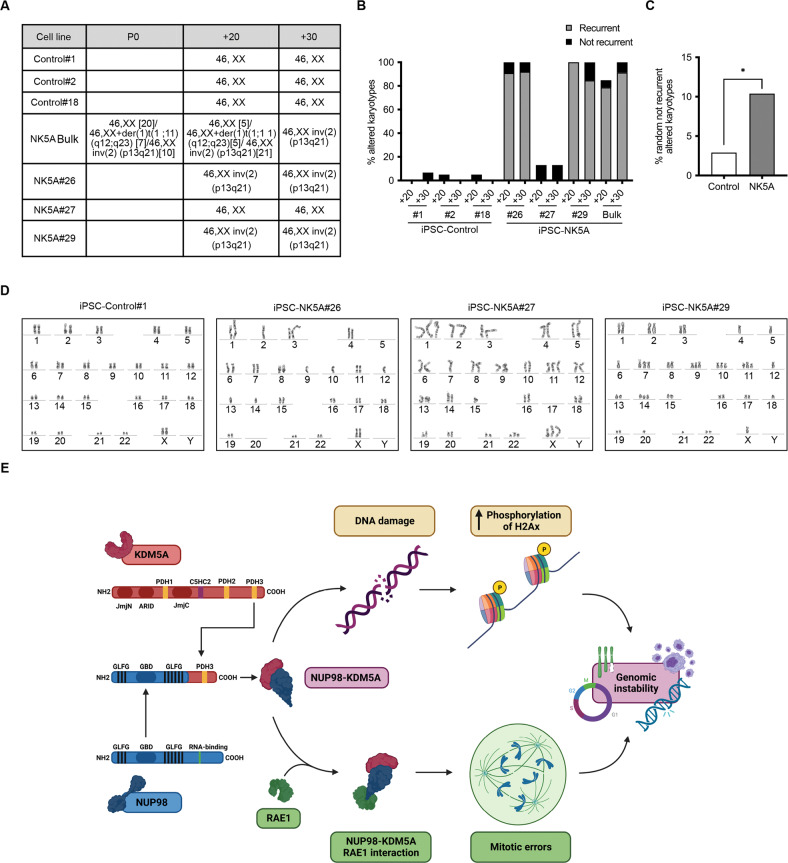
NUP98-JARI1A produces aberrant karyotypes. **A** Table showing the karyotypes of iPSC-control and NK5A clones at different passages. **B** Quantification of the altered karyotypes (recurrent and not recurrent) in the iPSC-control and iPSC-NK5A clones at two different passages. **C** Quantification of the random altered karyotypes comparing iPSC-control and iPSC-NK5A clones. iPSC-Control, *n* = 137. iPSC-NK5A, *n* = 163. **D** Examples of a normal karyotype from the iPSC-control#1 and aberrant karyotypes from iPSC-NK5A#26, NK5A#27 and NK5A#29. **E** Schematic representation of the proposed model. NUP98-KDM5A generates genomic instability through two complementary mechanisms: NUP98-KDM5A induces DNA damage and the accumulation of γ-H2AX, and the direct interaction of NUP98-KDM5A with RAE1 interfere with RAE1 activity during mitosis, inducing mitotic errors. *p < 0.05; Chi-square test applied (**C**).

To assess the consequences of chromosome missegregation we prepared metaphase spreads and analyzed the chromosomal status of NK5A-iPSC and control clones. Since the incidence of aneuploidy in the hPSCs increases significantly during time in culture [36], we analyzed bulk cultures after transduction (passage 0) and derived iPSC clones after 20 and 30 cell culture passages. The iPSC-NK5A founder line presented mosaic aneuploidy at passage 0, and over time in culture there was a selection of cells that presented the 46,XX inv (2)(p13q21) karyotype that overtook the culture after 30 passages (Fig. 7A). From the three iPSC-NK5A clones, two of them, NK5A#26 and NK5A#29, also displayed the altered 46,XX inv(2)(p13q21) karyotype, while the NK5A#27 clone conserved a normal karyotype after 30 passages (Fig. 7A). In contrast, all the control iPSC clones have normal diploid karyotype after 30 passages (Fig. 7A). Of note, the H9-NK5A clones, which expressed low levels NUP98-KDM5A, had a normal karyotype even after 30 passages (Supplementary Fig. 2C), suggesting that the role of NUP98-KDM5A in generating aneuploidy is dose dependent. While we were performing the cytogenetic analysis, we realized that the iPSC-NK5A clones exhibited diverse not-recurrent chromosomal abnormalities that were rare in control clones (Fig. 7B, D). Up to 10% of the randomly examined metaphase spreads displayed different (not-recurrent) chromosomal aberrations in iPSC-NK5A clones, (Fig. 7C), consistent with the percentage of cells that presented mitotic abnormalities (Fig. 5B).

In summary, our data indicate that NUP98-KDM5A expression in hPSC produces chromosome instability, increasing their predisposition to chromosome missegregation.

## Discussion

Pediatric AML is still an important medical challenge since the overall survival ranges between 20% to 70% depending on the AML subtype [1, 3]. There is limited progress on targeted therapies mainly due to its low frequency, the wide variety of genetic alterations associated to it and the scarce material available for studies, making very challenging to understand the biological basis of these group of diseases [4]. In many pediatric leukemia, genetic changes during development give rise to pre-leukemic clones before birth [6, 7, 37]. Since NUP98-KDM5A is restricted to infants and young AML patients [1, 11, 12], it is very likely that this fusion protein also appear prenatally.

Here, we show that NUP98-KDM5A expression in iPSC cells induced HIF1A stabilization and early transcriptomic changes in hypoxia-related genes, altered mitochondrial homeostasis and induced ROS-mediated DNA damage. A previous study has suggested that the early over-expression of hypoxia-responsive genes is essential for tumor evolution, as it induces chromosomal instability [38].

In the NUP98-KDM5A fusion, both fusion partners have the potential to regulate gene expression [17, 21, 39]. KDM5A can repress target genes by the removal of chromatin activation H3K4me3 marks [21]. It has been shown that NUP98-KDM5A interferes with the endogenous function of KDM5A, through the PHD domain of KDM5A moiety that recognizes the H3K4me3 marks [17]. In our transcriptomic analysis, half of the differentially expressed genes contain bivalent chromatin regions [40], suggesting that KDM5A moiety is acting as a dominant negative and promote the observed up-regulation of target genes. However, NUP98 can also regulate gene transcription [39] and it has been recently shown that NUP98 oncoproteins with different fusion partners displayed common targets, like CDK6 [41].

We show that expression of NUP98-KDM5A in iPSC produced a G2/M cell-cycle arrest and significantly increased ROS production and DNA damage. Studies in Philadelphia chromosome–positive acute lymphoblastic leukemia (ALL) showed that cells harboring the BCR-ABL fusion protein have elevated ROS levels that induced DSBs and genomic instability [42]. In BCR-ABL positive cells, the inhibition of proapoptotic mechanisms lead to malignant progression and drug resistance [43]. In our study, high levels of NUP98-KDM5A incremented the number of apoptotic cells, suggesting that the increase in DNA damage induce a strong apoptotic response as a protective mechanism against malignant progression. Therefore, it should be necessary to reduce the apoptotic response for malignant transformation of the NK5A-iPSC cells.

We further demonstrate that NUP98-KDM5A-expressing iPSC displayed several mitotic spindle defects, chromosome missegregation and aneuploudy, indicating that are genetically unstable. Some of the mitotic defects could be a consequence of unrepaired lesions of DSBs present at mitosis, as previous studies have demonstrated that extensive DDR during mitosis produce chromatin bridges and chromosome missegregation [29]. However is very likely that the direct interaction of NUP98-KDM5A with RAE1 play a major role in the observed genomic instability. During mitosis RAE1 form a complex with NUP98 that controls microtubule dynamics and mitotic progression [34, 44]. Deregulation of RAE1 expression lead to increased formation of multipolar spindles [45]. Therefore, in NUP98-KDM5A-expressing cells, interaction of the fusion protein with endogenous RAE1 might sequester RAE1 away from APC/CDH1 complex and interfere with the mitotic spindle assembly and the regulation of mitotic exit. Importantly, these results were reproducible in a hematopoietic cellular context, since the patient-derived cell line CHRF-288-11 harboring the NUP98-KDM5A fusion also showed increased DSDs, mitotic defects and direct interaction of NUP98-KDM5A fusion with RAE1 during mitosis.

The mechanisms leading to genomic instability induced by NUP98-KDM5A that we described here are consistent with findings in pediatric AML patients as increased DNA damage and chromosome aberrations have also been observed in a patient sample carrying the NUP98-NSD1 fusion [8]. Also, consistently with the fact that nearly all patients carrying NUP98-KDM5A protein display complex karyotypes [10, 13, 46], here we demonstrated that NUP98-KDM5A expression contributes to chromosome missegregation Overall, our results identify NUP98-KDM5A mechanisms to induce genomic instability that potentially can drive leukemia evolution (Fig. 7E).

## Materials and methods

### Cell lines

The human embryonic stem cell (hESC) line H9 (Wicell; Madison, WI, USA) and the induced pluripotent stem cell (iPSC) PBMC2-iPS4F8 [17] were cultured in 5% CO_2_ at 37 °C on Matrigel® Matrix (Corning; Corning, New York, USA). The cells were maintained using Essential 8 (E8) growth medium with daily media changes. For the clone generation, a limit dilution assay (0.5 cells/well) in 96-well plate was performed.

CHRF-288-11 cell line (kindly provided by Dr. Rolf Urbanus (University Medical Center Utrecht, The Netherlands) was cultured in suspension with DMEM (Biowest; Nuaillé, France) supplemented with 10% of FBS and 100 IU/mL of penicillin and streptomycin. When cells reached a density of 5 × 10^5^ cells/mL, cells were split every 2–3 days to a density of 1 × 10^5^ cells/mL. All cell lines were cultured in 5% CO_2_ at 37 °C in a humid incubator.

### Plasmid construction and lentiviral transduction

The cDNA of NUP98-KDM5A was obtained in a pMSCV vector (kindly provided by Dr David Allis; The Rockefeller University, New York, USA). The cDNA was sub-cloned using standard procedures into pRRL-EF1a-PGK-NEO vector (kindly provided by Prof. Trono, EPFL, Lausanne, Switzerland) for the expression in hPSCs. To generate the lentiviral vectors, HEK-293T cells were transfected with pRRL-EF1a-PGK-NEO (Control) or pRRL-EF1a-NUP98-KDM5A-PGK-NEO (NK5A) together with psPax2 (packaging vector) and VSV.G (envelope vector), (Addgene; Watertown, Massachusetts, USA) by standard calcium-phosphate transfection protocol. The supernatants were collected 48 h after transfection and used fresh supplemented with Polybrene Reagent (8 mg/mL, Sigma-Aldrich; St. Louis, Missouri, USA) and Y-27632 2HCl (10 μM, Selleckchem, Houston, TX, USA) to transduce hPSC (H9 and PBMC2-iPS4F8) in single cell suspension at the day of passage. After 2 days, the cells were selected with G418 (100 mg/mL; Invitrogen; Carlsbad, California, USA) for 5 days.

### RNA extraction, RT-PCR, and qPCR

Total RNA was extracted using the NuceloSpin® RNA kit (Macherey-Nagel; Düren, Germany) following manufacturer’s instructions. The cDNA was synthesized from RNA using the Transcriptor First Strand cDNA Synthesis Kit (Roche; Basel, Switzerland) following manufacturer’s instructions. Gene expression was analyzed by qPCR using Brilliant III Ultra-Fast SYBR® Green QPCR Master Mix (Agilent Technologies; Santa Clara, California, USA) in a 7900HT Fast Real-Time PCR System (Thermofisher; Waltham, Massachusetts, USA). The data was analyzed with the 2–ΔΔCT method using GAPDH to normalize.

### Western blot analysis

For total protein extraction, hPSCs were lysed with RIPA buffer (Sigma-Aldrich) containing protease inhibitor cocktail (Roche) and phosphatase inhibitors cocktail 2 and 3 (Sigma-Aldrich). For histone extraction we used the Histone extraction protocol for western blot from Abcam (Cambridge; UK) web page. Cell lysates were separated by molecular weight using SDS-polyacrylamide gels and transferred to polyvinylidene fluoride (PVDF) membranes. Protein was detected using the Odyssey Infrared Imaging System (Li-cor Biosciences; Lincoln, NE, USA). To detect KDM5A and NUP98-KDM5A was used the α-KDM5A antibody (ab70892, Abcam). Also used α-NUP98 (ab50610, Abcam), α-HIF1A (610959, BD Bioscience; San Jose, CA, USA), α-γ-H2AX (#9718, Cell signalling; Danvers, MA, USA). An α-β-Actin (A5441, Sigma-Aldrich) was used as a loading control for total protein extractions and α-H3 (ab1791, Abcam) was used as a loading control in histone extractions. Western blotting was carried out using standard procedures. Quantification of band intensity and normalization was carried out using ImageJ (NHI, Bethesda, Maryland, USA, https://imagej.nih.gov/ij).

### Detection of pluripotent markers by flow cytometry

hPSCs were dissociated using TrypLE™ Express (Invitrogen) and the single cell suspension was stained with PE-conjugated TRA-1–60 (12-8863-82, Invitrogen) or APC-conjugated SSEA-4 (560796, BD Bioscience) during 30 min for surface staining. Cells were washed with FACS buffer and stained with 7-aminoactinomycin D (7AAD) (BD Bioscience). Live cells identified by 7AAD exclusion were analyzed using FACSVerse® flow cytometer (BD Bioscience) and FlowJoTM (Ashland, OR, USA).

### Immunofluorescence

Cells were plated at low density in 24-well format containing a Matrigel® (Corning) treated dish. When desired confluence was reached, cells were washed with PBS and fixed with paraformaldehyde (Sigma-Aldrich) at 4% in PBS. The samples were blocked and permeabilised with 5% of BSA (Sigma-Aldrich) (w/v) in PBS and 0.3% of triton x-100 (Sigma-Aldrich) (v/v) for 5 min at room temperature. The washes were performed using PBS with 0.1% of BSA. The cell line CHRF-288-11 was fixed in suspension with paraformaldehyde at 2% in PBS for 20 min and permeabilized with PBS and 0.5% of triton x-100 for 5 min. The cells were dried with PBS over poly-L-lysine coated slides. The staining was performed overnight using a 5% BSA/PBS solution and the desired antibodies. To detect KDM5A and NUP98-KDM5A the same antibody was used, the α-KDM5A (ab70892, Abcam). Also used α-γH2AX (#9718, Cell Signaling Technology, Danvers, MA, USA), α-tubulin (sc-23948, Santa Cruz; Dallas, TX, USA), α-CDC20 (sc-13162, Santa Cruz) and α-MRNP41 (sc-374261, Santa Cruz). For mitochondria staining, Mitotracker 633 reagent (Invitrogen) was used at 200 nM for 40 min following manufacturer instructions. Then the dish was mounted and stained for DNA (DAPI) using VECTASHIELD® Antifade Mounting Media (Vector Laboratories, Burlingame, CA, USA).

### Growth curve

Pluripotent colonies were dissociated using TrypLE™ Express (Invitrogen) in single cell suspensions and plated. For growth curve 50,000 cells were plated in wells of 12-well plate supplemented with Y-27632 2HCl (10 μM, Selleckchem) and counted at day 1, 3 and 5. After 5 days of culture, cells were dissociated with TrypLE Express and counted. Again, 50,000 cells were replated and counted at day 8 and 10. The total number of cells at both days was the sum of the number of counted cells plus the number of cells at day 5 before the replating.

### Colony Forming Unit (CFU) assay

For CFU assays 100, 200 or 400 cells were plated in 6-well format with Y-27632 2HCl (10 μM, Selleckchem). At day 10, cells were fixed with 96% ethanol for 10 min and stained with Cristal violet (0.05%) for 30 min. The signal intensity of the colonies was detected with the Odyssey Infrared Imaging System (Li-cor Biosciences, Lincoln, NE, USA).

### EB differentiation

Cells were gently scraped off, centrifuged, resuspended into Essential 6 medium and plated over Ultra-Low Attachment Well Plate Corning® (Merck; Darmstadt, Germany). (Corning) and embryo bodies were formed spontaneously.

### Cell cycle analysis

Cells were dissociated using TrypLE™ Express (Invitrogen) and fixed using cold 70% ethanol and frozen at −20 °C. Cells were washed with PBS1x and stained with propidium iodide (50 μg/mL) (Sigma-Aldrich) and RNAse A (100 μg/mL) (Sigma-Aldrich). The samples were analyzed in the FACSVerse® flow cytometer (BD Bioscience, San Jose, CA, USA). The cell cycle profiles were analyzed using the Modfit software (Verity Software, Topsham, ME, USA).

### Apoptosis analysis

Cells were dissociated using TrypLE™ Express (Invitrogen) and counted. Annexin-V apoptosis detection kit (BD Biosciences) was used following manufacturer’s instructions. The samples were analyzed in the FACSVerse® flow cytometer (BD Bioscience) and FlowJo™ Software (Ashland, OR, USA).

### ROS levels analysis

We used the DCFDA / H2DCFDA - Cellular ROS Assay Kit (ab113851, Abcam, Cambridge, UK) for detection of reactive species of oxygen, following the manufacturer’s instructions.

### Alkaline phosphatase assay

Colonies were assayed for phosphatase alkaline enzymatic activity using a commercial detection kit (Sigma-Aldrich) following manufacturer’s instructions.

### Statistical analysis

All data is expressed as mean ± standard error of the mean. For statistical comparisons between two groups we performed the Two-tailed unpaired Student’s t-student test. For statistical comparisons between more than two groups or conditions, the two-way ANOVA test was applied. The chi-squared test was applied for the percentage of positive cells for γ-H2AX, aberrant mitosis and aberrant karyotypes. Mann-Whitney test was applied for the comparison of immunofluorescence signal of Mitotracker 633 and H2AX in mitosis. The data was considered significative when *p* value < 0.05. The number of biological replicates (n) is specified in each figure legend. The tests applied are two-sided and they have adjustment for multiple comparisons.

## Supplementary information

Materials and methods suppl-final

Supplementary figure legend -Final

Supplementary table 1.

Supplementary Figure 1

Supplementary Figure 2

Supplementary Figure 3

Supplementary Figure 4

Original WB ACTIN Figure 1D

Original WB KDM5A Figure 1D

Original WB NUP98 Figure 1G

Original WB ACTIN Figure 1G

Original WB KDM5A Figure 1G

Original WB ACTIN Figure 3 E

Original WB HIF1A Figure 3 E

Original WB H3 Figure 4E

Original WB H2AX Figure 4E

Original WB KDM5A Figure 5E

Original WB RAE1 Figure 5E

Original WB KDM5A Figure 6F

Original WB RAE1 Figure 6F

aj-checklist- CDDIS-22-2616-Final

## Data Availability

The RNA-seq data have been deposited in the NCBI Gene Expression Omnibus under accession number GSE224499.

## References

[1] Bolouri H, Farrar JE, Triche T, Ries RE, Lim EL, Alonzo TA (2018). The molecular landscape of pediatric acute myeloid leukemia reveals recurrent structural alterations and age-specific mutational interactions. Nat Med.

[2] Zwaan CM, Kolb EA, Reinhardt D, Abrahamsson J, Adachi S, Aplenc R (2015). Collaborative efforts driving progress in pediatric acute myeloid leukemia. J Clin Oncol.

[3] Elgarten CW, Aplenc R (2020). Pediatric acute myeloid leukemia: Updates on biology, risk stratification, and therapy. Curr Opin Pediatr.

[4] Lonetti A, Pession A, Masetti R (2019). Targeted therapies for pediatric AML: gaps and perspective. Front Pediatr.

[5] McNeer NA, Philip J, Geiger H, Ries RE, Lavallée VP, Walsh M (2019). Genetic mechanisms of primary chemotherapy resistance in pediatric acute myeloid leukemia. Leukemia [Internet]..

[6] Greaves MF, Maia AT, Wiemels JL, Ford AM (2003). Leukemia in twins: lessons in natural history. Blood.

[7] Greaves MF, Wiemels J (2003). Origins of chromosome translocations in childhood leukaemia. Nat Rev Cancer.

[8] Bisio V, Zampini M, Tregnago C, Manara E, Salsi V, Di Meglio A (2017). NUP98-fusion transcripts characterize different biological entities within acute myeloid leukemia: a report from the AIEOP-AML group. Leukemia.

[9] Struski S, Lagarde S, Bories P, Puiseux C, Prade N, Cuccuini W (2017). NUP98 is rearranged in 3.8% of pediatric AML forming a clinical and molecular homogenous group with a poor prognosis. Leukemia [Internet].

[10] van Zutven LJCM, Onen E, Velthuizen SCJM, van Drunen E, von Bergh ARM, van den Heuvel-Eibrink MM (2006). Identification of NUP98 abnormalities in acute leukemia: JARID1A (12p13) as a new partner gene. Genes Chromosomes Cancer [Internet].

[11] Noort S, Wander P, Alonzo TA, Smith J, Ries RE, Gerbing RB, et al. The clinical and biological characteristics of NUP98-KDM5A in pediatric acute myeloid leukemia. Haematologica. 2020. haematol.2019.236745 10.3324/haematol.2019.236745 PMC7849578 32381579

[12] De Rooij JDE, Masetti R, Van Den Heuvel-Eibrink MM, Cayuela JM, Trka J, Reinhardt D (2016). Recurrent abnormalities can be used for risk group stratification in pediatric AMKL: a retrospective intergroup study. Blood..

[13] Hara Y, Shiba N, Ohki K, Tabuchi K, Yamato G, Park MJ (2017). Prognostic impact of specific molecular profiles in pediatric acute megakaryoblastic leukemia in non-Down syndrome. Genes Chromosomes Cancer.

[14] De Rooij JDE, Branstetter C, Ma J, Li Y, Walsh MP, Cheng J (2017). Pediatric non-Down syndrome acute megakaryoblastic leukemia is characterized by distinct genomic subsets with varying outcomes. Nat Genet [Internet].

[15] Merkle FT, Eggan K (2013). Modeling human disease with pluripotent stem cells: from genome association to function. Cell Stem Cell [Internet].

[16] Patterson M, Chan DN, Ha I, Case D, Cui Y, Handel B, Van (2012). Defining the nature of human pluripotent stem cell progeny. Cell Res.

[17] Wang GG, Song J, Wang Z, Dormann HL, Casadio F, Li H (2009). Haematopoietic malignancies caused by dysregulation of a chromatin-binding PHD finger. Nature [Internet].

[18] Takeda A, Goolsby C, Yaseen NR (2006). NUP98-HOXA9 induces long-term proliferation and blocks differentiation of primary human CD34+ hematopoietic cells. Cancer Res.

[19] Pasini D, Hansen KH, Christensen J, Agger K, Cloos PAC, Helin K (2008). Coordinated regulation of transcriptional repression by the RBP2 H3K4 demethylase and polycomb-repressive complex 2. Genes Dev.

[20] Chen J, Liang X, Zhang S, Wang S, Garcia SP, Yan P, et al. Two faces of bivalent domain regulate VEGFA responsiveness and angiogenesis. Cell Death Dis [Internet]. 2020;11. 10.1038/s41419-020-2228-3 10.1038/s41419-020-2228-3 PMC6992747 32001672

[21] Voigt P, Tee WW, Reinberg D (2013). A double take on bivalent promoters. Genes Dev.

[22] Salceda S, Caro J (1997). Hypoxia-inducible factor 1α (HIF-1α) protein is rapidly degraded by the ubiquitin-proteasome system under normoxic conditions. Its stabilization by hypoxia depends on redox-induced changes. J Biol Chem.

[23] Schödel J, Oikonomopoulos S, Ragoussis J, Pugh CW, Ratcliffe PJ, Mole DR (2011). High-resolution genome-wide mapping of HIF-binding sites by ChIP-seq. Blood..

[24] Lachmann A, Xu H, Krishnan J, Berger SI, Mazloom AR, Ma’ayan A (2010). ChEA: transcription factor regulation inferred from integrating genome-wide ChIP-X experiments. Bioinformatics..

[25] Matys V, Kel-Margoulis OV, Fricke E, Liebich I, Land S, Barre-Dirrie A (2006). TRANSFAC and its module TRANSCompel: transcriptional gene regulation in eukaryotes. Nucleic Acids Res..

[26] Guzy RD, Hoyos B, Robin E, Chen H, Liu L, Mansfield KD (2005). Mitochondrial complex III is required for hypoxia-induced ROS production and cellular oxygen sensing. Cell Metab.

[27] Patten DA, Lafleur VN, Robitaille GA, Chan DA, Giaccia AJ, Richard DE. Hypoxia-inducible factor-1 activation in nonhypoxic conditions: the essential role of mitochondrial-derived reactive oxygen species. Gutkind JS, editor. Mol Biol Cell [Internet]. 2010;21:3247–57. 10.1091/mbc.e10-01-0025. 10.1091/mbc.E10-01-0025 PMC2938389 20660157

[28] Srinivas US, Tan BWQ, Vellayappan BA, Jeyasekharan AD (2019). ROS and the DNA damage response in cancer. Redox Biol [Internet].

[29] Bakhoum SF, Kabeche L, Compton DA, Powell SN, Bastians H (2017). Mitotic DNA damage response: at the crossroads of structural and numerical cancer chromosome instabilities. Trends in Cancer.

[30] Rieder CL (2011). Mitosis in vertebrates: the G2/M and M/A transitions and their associated checkpoints. Chromosom Res.

[31] Salsi V, Fantini S, Zappavigna V (2016). NUP98 fusion oncoproteins interact with the APC/CCdc20 as a pseudosubstrate and prevent mitotic checkpoint complex binding. Cell Cycle [Internet].

[32] Salsi V, Ferrari S, Gorello P, Fantini S, Chiavolelli F, Mecucci C (2014). NUP98 fusion oncoproteins promote aneuploidy by attenuating the mitotic spindle checkpoint. Cancer Res.

[33] Mi H, Muruganujan A, Thomas PD (2013). PANTHER in 2013: modeling the evolution of gene function, and other gene attributes, in the context of phylogenetic trees. Nucleic Acids Res.

[34] Jeganathan KB, Malureanu L, Van Deursen JM (2005). The Rae1-Nup98 complex prevents aneuploidy by inhibiting securin degradation. Nature..

[35] Funasaka T, Nakano H, Wu Y, Hashizume C, Gu L, Nakamura T (2011). RNA export factor RAE1 contributes to NUP98-HOXA9-mediated leukemogenesis. Cell Cycle.

[36] Barbaric I, Biga V, Gokhale PJ, Jones M, Stavish D, Glen A (2014). Time-lapse analysis of human embryonic stem cells reveals multiple bottlenecks restricting colony formation and their relief upon culture adaptation. Stem Cell Reports [Internet].

[37] Li Z, Godinho FJ, Klusmann JH, Garriga-Canut M, Yu C, Orkin SH (2005). Developmental stage-selective effect of somatically mutated leukemogenic transcription factor GATA1. Nat Genet.

[38] Jing A, Vizeacoumar FS, Parameswaran S, Haave B, Cunningham CE, Wu Y (2018). Expression-based analyses indicate a central role for hypoxia in driving tumor plasticity through microenvironment remodeling and chromosomal instability. npj Syst Biol Appl.

[39] Liang Y, Franks TM, Marchetto MC, Gage FH, Hetzer MW (2013). Dynamic association of NUP98 with the human genome. PLoS Genet.

[40] Court F, Arnaud P (2017). An annotated list of bivalent chromatin regions in human ES cells: a new tool for cancer epigenetic research. Oncotarget..

[41] Schmoellerl J, Barbosa IAM, Eder T, Brandstoetter T, Schmidt L, Maurer B (2020). CDK6 is an essential direct target of NUP98 fusion proteins in acute myeloid leukemia. Blood..

[42] Nowicki MO, Falinski R, Koptyra M, Slupianek A, Stoklosa T, Gloc E (2004). BCR/ABL oncogenic kinase promotes unfaithful repair of the reactive oxygen species-dependent DNA double-strand breaks. Blood..

[43] Slupianek A, Hoser G, Majsterek I, Bronisz A, Malecki M, Blasiak J (2002). Fusion tyrosine kinases induce drug resistance by stimulation of homology-dependent recombination repair, prolongation of G2/M phase, and protection from apoptosis. Mol Cell Biol.

[44] Cross MK, Powers MA (2011). Nup98 regulates bipolar spindle assembly through association with microtubules and opposition of MCAK. Mol Biol Cell.

[45] Wong RW, Blobel G, Coutavas E (2006). Rae1 interaction with NuMA is required for bipolar spindle formation. Proc Natl Acad Sci USA.

[46] De Rooij JDE, Branstetter C, Ma J, Li Y, Walsh MP, Cheng J (2017). Pediatric non-Down syndrome acute megakaryoblastic leukemia is characterized by distinct genomic subsets with varying outcomes. Nat Genet.

