# Supplementary material for: The pediatric leukemia oncoprotein NUP98-KDM5A induces genomic instability that may facilitate malignant transformation
Source: Cell Death Dis. 2023 Jun 10;14(6):357. doi: 10.1038/s41419-023-05870-5 (PMC10257648; doi:10.1038/s41419-023-05870-5)
Supplement: Supplementary file 1 — Materials and methods suppl-final [file 41419_2023_5870_MOESM1_ESM.doc]

## SUPPLEMENTARY MATERIALS & METHODS

## Immunofluorescence analysis

## The immunofluorescence preparations were analysed in a Zeiss LSM 710 confocal microscope (Zeiss; Oberkochen, Germany) or a Zeiss Axio Imager A.1 fluorescence microscope (Zeiss)

## For confocal microscopy ZEISS the objective used were the ZEISS Plan-Apochromat 63x 1.40 NA Oil DIC M27 and the photomultiplier Tubes (PMTs) Point Detector. The filter model was the main Beam Splitters (MBS). DAPI: MBS InVis 405 nm. Detection Range: 410-487 nm. A488: MBS 488/594 nm. Detection Range: 493-540 nm. A555: MBS 458/543 nm. Detection Range: 548-697 nm. A633: MBS 488/543/633 nm. Detection range: 638-747 nm. The Batch Number of the equipment is 2502000526 and the images were acquired with the software: ZEN 2010 Version 6.0*).*

## For the images the Look Up Table (LUTs) are: DAPI: Blue. A488: Green. A555: Red. A633: Green. Quantitative map between the LUT and the bitmap: Bit Depth: 16-Bits. Range: 0-65535 Grey Levels. The LUT is linear in all images and the resolution size of the images are 512 pixels x 512 pixels.

## Images were analysed and adjusted for brightness and contrast using ImageJ. To determine the R coefficient of overlapping, the distance between the signals of KDM5A and RAE1 were measured with ZEISS ZEN Black Microscopy Software using the co-localization module.

## RNAseq

## Total RNA extraction of the three iPSC-control clones (iPS4F8 Control#1, #2 and #18) and the three NK5A-expressing iPSCs clones (iPS4F8 NK5A#26, NK5A#27 and NK5A#29) was performed using the NuceloSpin® RNA kit (Macherey-Nagel) following manufacturer’s instructions. The RNA was quantified, and the integrity checked by the Bioanalyzer RNA Nano (Agilent Technologies; Santa Clara, California, USA) giving adequate values of RIN (RNA Integrity Number; minimum RIN of 7). The TruSeq Stranded mRNA (Illumina; San Diego, California, USA) was used following the manufacturer’s instructions. The samples were sequenced in a Nextseq 500 (Illumina).

## Bioinformatics analysis

## Once we had the sequences, first a quality control was carried out using FastQC ([**http://www.bioinformatics.babraham.ac.uk/projects/fastqc**](http://www.bioinformatics.babraham.ac.uk/projects/fastqc)). Next, in order to calculate gene expression, the alignment of RNA was performed using RSEM software (1) with default parameters and using the GRCh38.p13 as reference genome. Finally, the differential expression analysis followed the standard pipeline of DESeq2 package. We considered as differentially expressed genes those with p-value < 0.05 and log2FoldChange > 1 and < -1. Heatmaps shows their TMM normalized expression and they were designed using pheatmap ([**https://cran.r-project.org/web/packages/pheatmap/index.html**](https://cran.r-project.org/web/packages/pheatmap/index.html)). The vulcano plot is done with R's package EnhancedVolcano ([**https://github.com/kevinblighe/EnhancedVolcano**](https://github.com/kevinblighe/EnhancedVolcano)), it represents the Fold Change in Log2 and the mean of gene expression in Log2 per sample class those differentially expressed are highlighted in red.

## The gene set enrichment analysis (GSEA) was performed using GSEA software. (2) The genes were ranked according to the Wald statistic obtained in the differential expression analysis. We compared our genes to the gene sets collection of MSigDB (2) CGP: chemical and genetic perturbations and significant gene sets (adjusted pvalue < 0.05) were selected for further analysis. From these results we selected the leading-edge genes of the top 8 enriched gene sets, related to hypoxia and HIF1A/HIF2A targets, to represent their expression values in a heatmap. Principal Component Analysis was done using the TMM normalized counts via R's base libray ([**https://www.r-project.org/**](https://www.r-project.org/) ) prcomp function and displayed with rgl package.

## Karyotypes analysis

## For cytogenetic analysis, the cells are incubated in E8 medium supplemented with 0.1 mg/mL of colcemide (Sigma-Aldrich) for 4 hours. The cytoplasm is removed using a hypotonic solution of KCl (0.075 M), and the nuclei are fixed with methanol:acetic in a 3:1 ratio (vol/vol). Finally, the metaphases are fixed in slides. G bands are made with trypsin-Whrigh (GTG) and a minimum of 20 metaphases are analysed for each cell line, 30 are analysed if it has chromosomal alterations, assigning a karyotype formula according to the International System of Human Cytogenetic Nomenclature (ISCN) 2016. The karyotypes are analysis using a microscope Leica DM5500 (Leica) and the Ikaros Karyotyping System (Metasystems; Heidelberg, Germany).

## Mitotic arrest

## The cells were arrested in mitosis using nocodazole (Sigma-Aldrich) at 0.1 ug/mL for 16 hours. Media was changed 1 hour before the collection of the cells. The percentage of cells in G2/M was confirmed by propidium iodide staining and flow cytometry using the FACSVerse® flow cytometer (BD Bioscience).

## Co-inmunoprecipitation

## Cells arrested with nocodazole were processed following the protocol described previously.(3) Briefly, fresh cells were lysed using de IP-buffer (50 mM Tris-HCl [pH 7.5), 150 mM NaCl, 1 mM EDTA, 0.5% NP-40, 10% glycerol and 1.5 mM MgCl_2_). After 30 minutes of incubation at 4°C and rotation, a centrifugation at 13,000 rpm, 10 minutes at 4°C was applied. 1 mg of protein was incubated with 4 μg of α-KDM5a and 25 μL of Dynabeads^TM^ Protein G (Thermofisher) in a total volume of 500 μL at 4°C and rotation overnight. The same amount of unspecific IgG was used as a control. After 3 washes in the same IP-buffer using the DynaMag^TM^ the elution was performed using 60 μL of Nupage buffer (2x, with 50 mM DTT) and heated at 70°C for 15 minutes. The Co-IP was confirmed by western blotting using the same α-KDM5a to prove the precipitation of KDM5A and NUP98-KDM5A. The interaction with RAE1 was confirmed using the antibody α-MRNP41 (sc-374261, Santa Cruz).

## Proteomics analysis

## Samples were prepared and analyzed in Proteomics Facility at Research Support Central Service at University of Cordoba. Protein extracts were cleaned-up in 1D SDS-PAGE at 10% of polyacrilamyde. Protein bands digestion was performed by addition of Trypsin (Promega; Madison, Wisconsin, USA), 12.5 ng/μL of enzyme in 25 mM AB and incubated at 37 ºC temperature overnight. Protein digestion was stopped by addition of trifluoroacetic acid at 1% final concentration. Nano LC was performed in Dionex Ultimate 3,000 nano UPLC (Thermofisher) with a C18 75 μm x 50 Acclaim Pepmam column (Thermofisher).

## The raw data was processed using Proteome Discoverer (version 2.1.0.81, Thermofisher). MS2 spectra were searched with SEQUEST engine against a database of Homo sapiens (www.uniprot.org). Peptide spectral matches (PSM) were validated using percolator based on q-values at a 1% FDR. With proteome Discoverer, peptide identifications were grouped into proteins according to the law of parsimony and filtered to 1% FDR.

## The analysis of detected peptides was carried out using R comparing first the pulldown of the α-KDM5A against the IgG of the sample. Only peptides present in the pulldown of the α-KDM5A of iPSC-NK5A#29 and absent in iPSC-Control#1 were considered fusion protein interactor candidates. Gene ontology (GO) of Nup98-KDM5A 18 candidate interactors was performed by the Protein Analysis Through Evolutionary Relationships (PANTHER) Classification System (version 15.0.), available online (www.pantherdb.org). We used the overrepresentation test using the default parameters: Fisher’s exact test, with FDR correction for multiple testing and the background reference list: Homo sapiens whole genome. The Heatmap was generated using the R function heatmap.2.

## Hematopoietic differentiation

## The differentiation from hPSC to hematoendothelial progenitors was performed using the STEMdiffTM Hematopoietic kit (STEMCELL® Tehcnologies, Canada, Ref: 05310) following the manufacturer’s instructions. The differentiations were analyzed by flow cytometry at day 3 and day 8.

## H2AX detection by flow cytometry

## NK5A-iPSCs and Control-iPSCs at day 8 of hematopoietic differentiation, Cord Blood and the CHRF-288-11 cell line were fixed and permeabilized using the PERM&FIX solution (Thermofisher, Ref: GAS003) following the manufacturer’s instructions and stained for CD34 and H2AX for flow cytometry. From the CD34^+^ population the expression levels of H2AX were analysed.

## ANTIBODIES USED IN THIS STUDY

| **Antigen** | **Application** | **Fluorophore** | **Brand** | **Reference** |
| --- | --- | --- | --- | --- |
| KDM5A | WB, IF, IP | - | Abcam | ab70892 |
| NUP98 | WB | - | Abcam | ab50610 |
| TRA-1-60 | Flow cytometry | PE | eBioscience | 12-8863-82 |
| SSEA-4 | Flow cytometry | Alexa Fluor® 647 | BD Pharmingen | 560796 |
| OCT3/4 | Flow cytometry | - | BD Pharmingen | 611203 |
| CD31 | Flow cytometry | V450 | BD Biosciences | #561653 |
| CD34 | Flow cytometry | PE-Cy7 | BD Biosciences | #555459 |
| CD43 | Flow cytometry | APC | BD Biosciences | #560198 |
| CD56 | Flow cytometry | PE | BD Biosciences | #561903 |
| HIF1α | WB | - | BD Bioscience | 610959 |
| γ-H2AX | WB, IF, Flow cytometry | - | Cell signalling | #9718 |
| α-TUBULIN | IF | - | Santa Cruz | sc-23948 |
| α-mrnp41 (RAE1) | WB, IF | - | Santa Cruz | sc-374261 |
| CDC20 | IF | - | Santa Cruz | sc-13162 |
| H3 | WB | - | Abcam | ab1791 |
| β-Actin | WB | - | Sigma-Aldrich | A5441 |

**PRIMERS USED IN THIS STUDY**

| **Gene** | **Primer Sequence** | |
| --- | --- | --- |
| *NUP98-KDM5A* | Forward | AAGATTGGAGGGCCTCTTGG |
|  | Reverse | GTGGTTTCTCTTCCATGCTGTC |
| *NUP98* | Forward | CCATCTATGGATGACCTTGCTAAA |
|  | Reverse | TCCGACCAATAGTGAAAATCAGAGA |
| *KDM5A* | Forward | TGGATTTCCGGTGAAGGAT |
|  | Reverse | TCCAGGACAGGCATGTTATTC |
| *POU5F1* | Forward | AGTGAGAGGCAACCTGGAGA |
|  | Reverse | ACACTCGGACCACATCCTTC |
| *SOX2* | Forward | TCAGGAGTTGTCAAGGCAGAGAAG |
|  | Reverse | CTCAGTCCTAGTCTTAAAGAGGCAGC |
| *NANOG* | Forward | TGCAGTTCCAGCCAAATTCTC |
|  | Reverse | CCTAGTGGTCTGCTGTATTACATTAAGG |
| *APLNR* | Forward | AAAGCCGACTTGCAAAACC |
|  | Reverse | TGGACCAAATTGACCCCTAC |
| *MIXL1* | Forward | GGATCCAGGTATGGTTCCAG |
|  | Reverse | GGAGCACAGTGGTTGAGGAT |
| *OTX2* | Forward | GACCCGGTACCCAGACATC |
|  | Reverse | GCTCTTCGATTCTTAAACCATACC |
| *FOXA1* | Forward | GTGGCTCCAGGATGTTAGGA |
|  | Reverse | GCCTGAGTTCATGTTGCTGA |
| *GAPDH* | Forward | TGCACCACCAACTGCTTAGC |
|  | Reverse | GGCATGGACTGTGGTCATGAG |

1. Li B, Dewey CN. RSEM: accurate transcript quantification from RNA-Seq data with or without a reference genome. BMC Bioinformatics [Internet]. 2011 Dec 4;12(1):323. Available from: https://bmcbioinformatics.biomedcentral.com/articles/10.1186/1471-2105-12-323

2. Subramanian A, Tamayo P, Mootha VK, Mukherjee S, Ebert BL, Gillette MA, et al. Gene set enrichment analysis: A knowledge-based approach for interpreting genome-wide expression profiles. Proc Natl Acad Sci U S A. 2005;102(43):15545–50.

3. Beringer M, Pisano P, Di Carlo V, Blanco E, Chammas P, Vizán P, et al. EPOP Functionally Links Elongin and Polycomb in Pluripotent Stem Cells. Mol Cell. 2016;64(4):645–58.
