# Supplementary material for: The pediatric leukemia oncoprotein NUP98-KDM5A induces genomic instability that may facilitate malignant transformation
Source: Cell Death Dis. 2023 Jun 10;14(6):357. doi: 10.1038/s41419-023-05870-5 (PMC10257648; doi:10.1038/s41419-023-05870-5)
Supplement: Supplementary file 2 — Supplementary figure legend -Final [file 41419_2023_5870_MOESM2_ESM.doc]

**Supplementary Figure 1**

**Characterization of hPSC founder lines expressing NUP98-KDM5A.** (A) Quantitative real time polymerase chain reaction (qRT-PCR) analysis showing the mRNA expression of pluripotency genes in iPSC-Control and NK5A, n=3. (B) qRT-PCR analysis showing the mRNA expression of pluripotency genes in H9-Control and NK5A, n=3. (C) Flow cytometry analysis of the pluripotent markers Tra-1-60 and SSEA4 in iPSC- control, iPSC-NK5A, H9-control and H9-NK5A. (D) Pictures of positive colonies for alkaline phosphatase activity in iPSC-control and NK5A lines. Scale bar = 150μm. (E) Pictures of positive colonies for alkaline phosphatase activity in H9- control and NK5A lines. Scale bar = 150μm. (F) qRT-PCR analysis showing the mRNA expression of endogenous *NUP98* and *KDM5A* in iPSC-control and NK5A, n=3. (G) qRT- PCR analysis showing the mRNA expression of endogenous *NUP98* and *KDM5A* in H9- control and NK5A, n=3. (H) Representative image of the immunofluorescence detecting endogenous KDM5A protein and NUP98-KDM5A fusion protein in H9-control and NK5A line. Scale bar = 10μm. (I) Screening of the *NUP98-KDM5A* mRNA expression by qRT-PCR in the generated iPSC and H9 clones. (J) qRT-PCR analysis showing the expression of the *NUP98-KDM5A* in the iPSC clones. (K) Graphic representation of the NUP98-KDM5A protein levels in the iPSC clones, n=2. Data in plots indicate mean ± SEM.

**Supplementary Figure 2**

**Characterization of the H9-NK5A clonal lines.** (A) Colony forming units (CFU) assay of the H9-control and H9-NK5A expressing clones, using 100, 200 and 400 initial cells, n=3. (B) Cell cycle profile using propidium iodide showing the percentage of cells in each cell cycle phase of the H9-control and H9-NK5A clones. (C) Table showing the karyotypes of H9-control and NK5A clones at different passages. Data in plots indicate mean ± SEM.

**Supplementary Figure 3**

(A) Dot plot of Mean of gene expression *vs* fold-change between iPSC-NK5A and control clones. In red the genes with an adjusted p-value < 0.05. (B) GSEA enrichment plots for relevant gene sets.

**Supplementary Figure 4**

**Double-immunofluorescence staining for NUP98-KDM5A and CDC20 during mitosis.** (A) Representative images of immunofluorescence of each cell cycle phase of iPSC-C#2 and NK5A#29. In red, KDM5A, in green, CDC20 and in blue, DNA. Scale bar = 10μm.
