# Supplementary figures and images for: The pediatric leukemia oncoprotein NUP98-KDM5A induces genomic instability that may facilitate malignant transformation
Source: Cell Death Dis. 2023 Jun 10;14(6):357. doi: 10.1038/s41419-023-05870-5 (PMC10257648; doi:10.1038/s41419-023-05870-5)

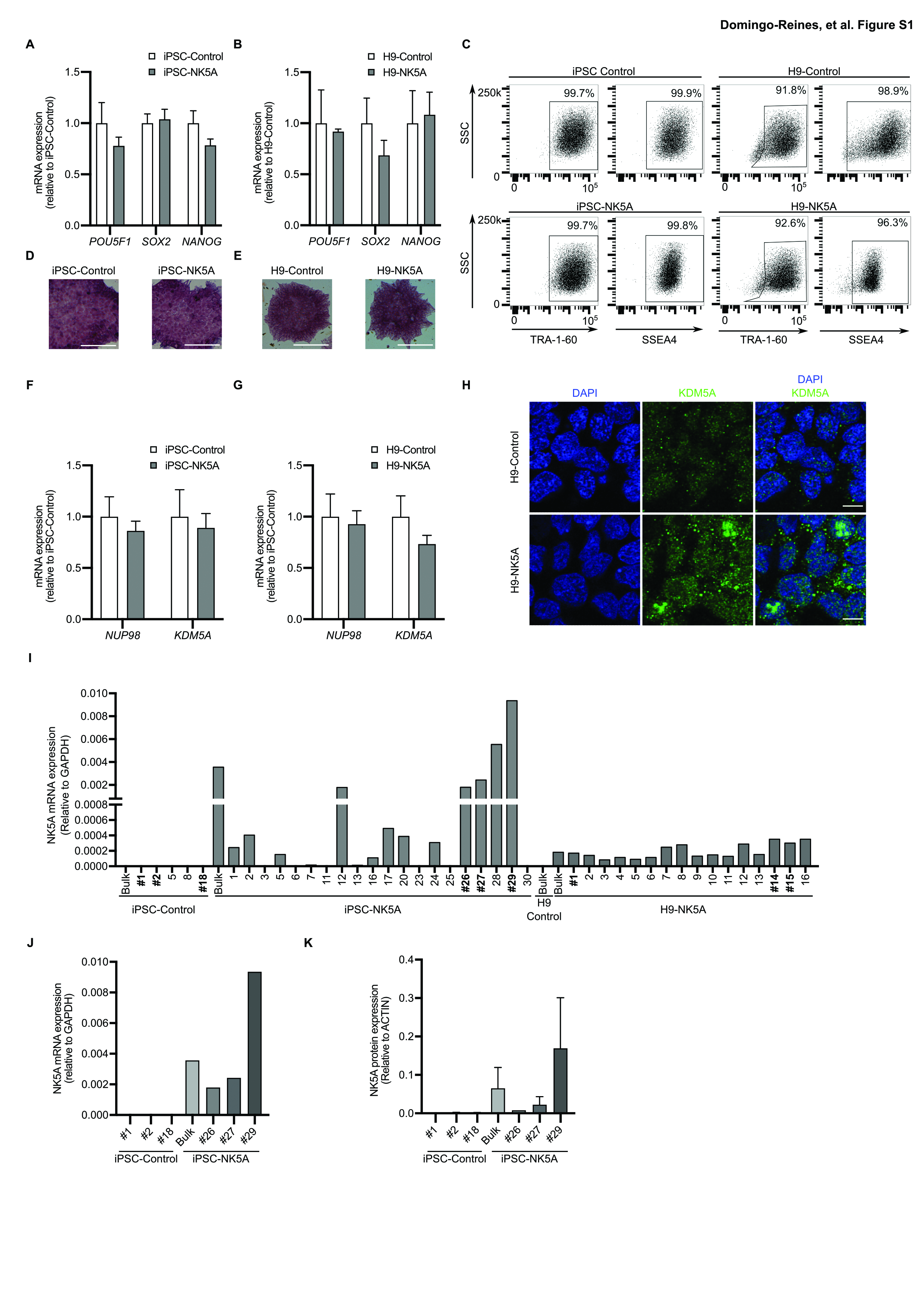

Supplement: Supplementary file 4 — Supplementary Figure 1 [file 41419_2023_5870_MOESM4_ESM.tif]

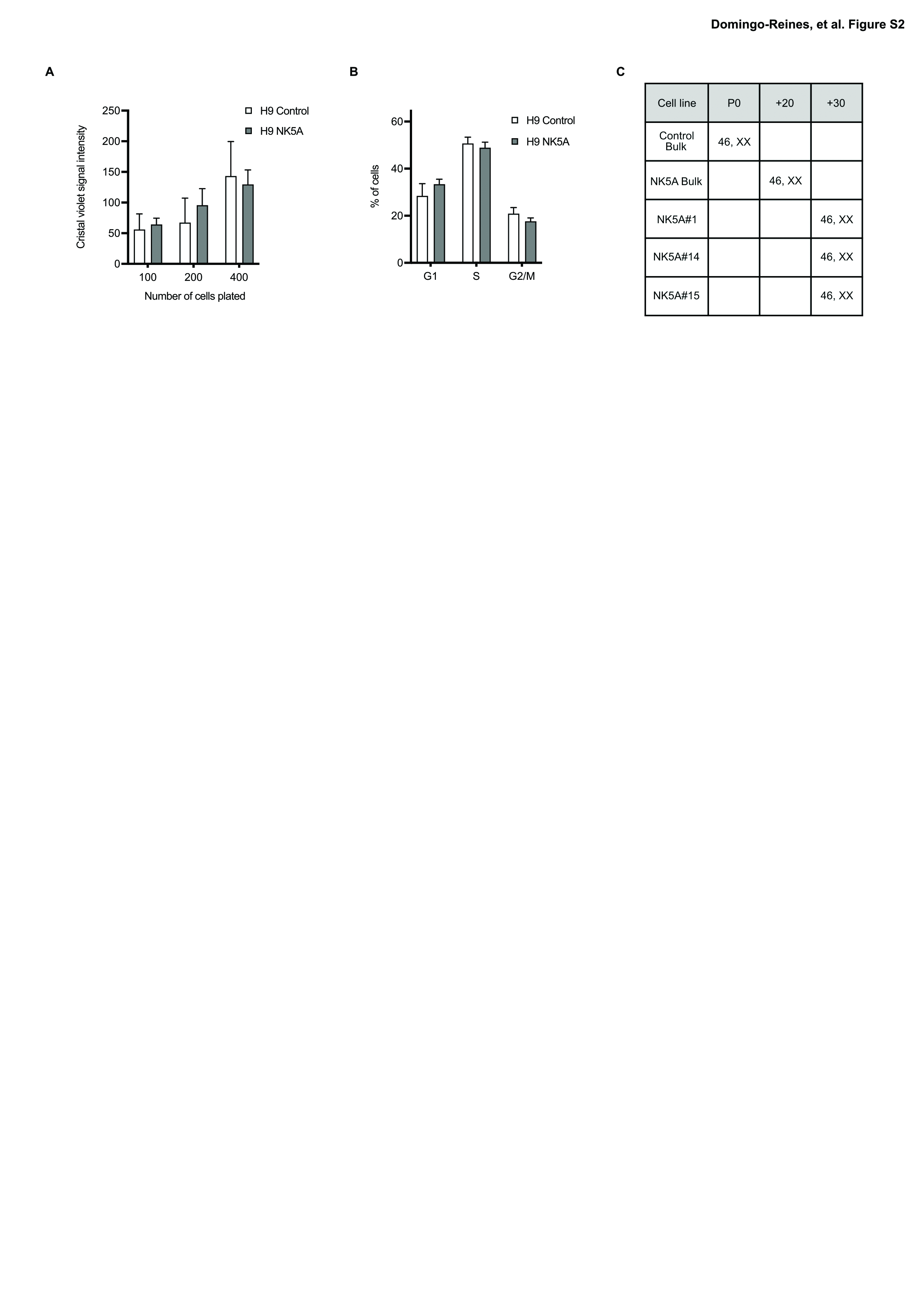

Supplement: Supplementary file 5 — Supplementary Figure 2 [file 41419_2023_5870_MOESM5_ESM.tif]

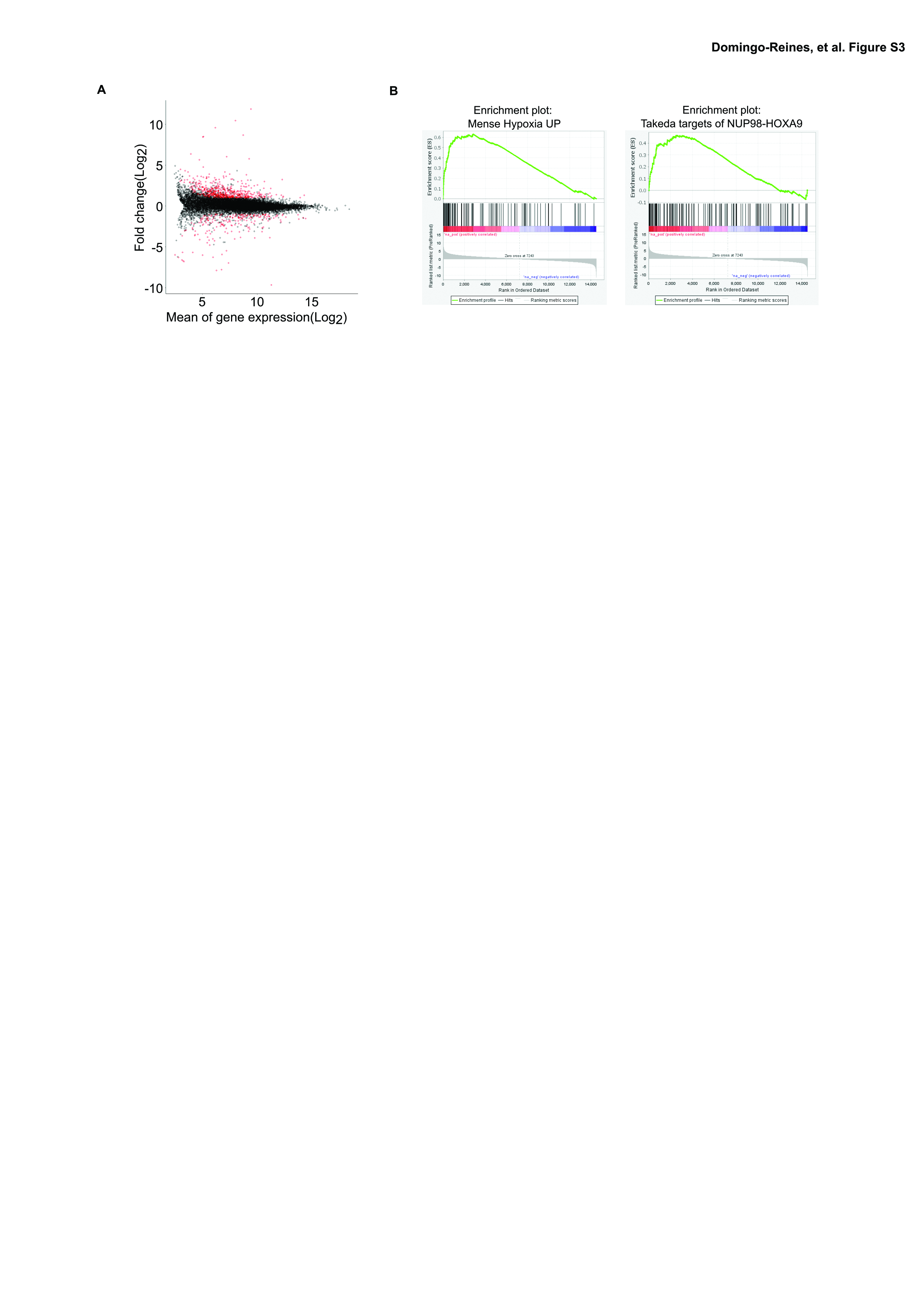

Supplement: Supplementary file 6 — Supplementary Figure 3 [file 41419_2023_5870_MOESM6_ESM.tif]

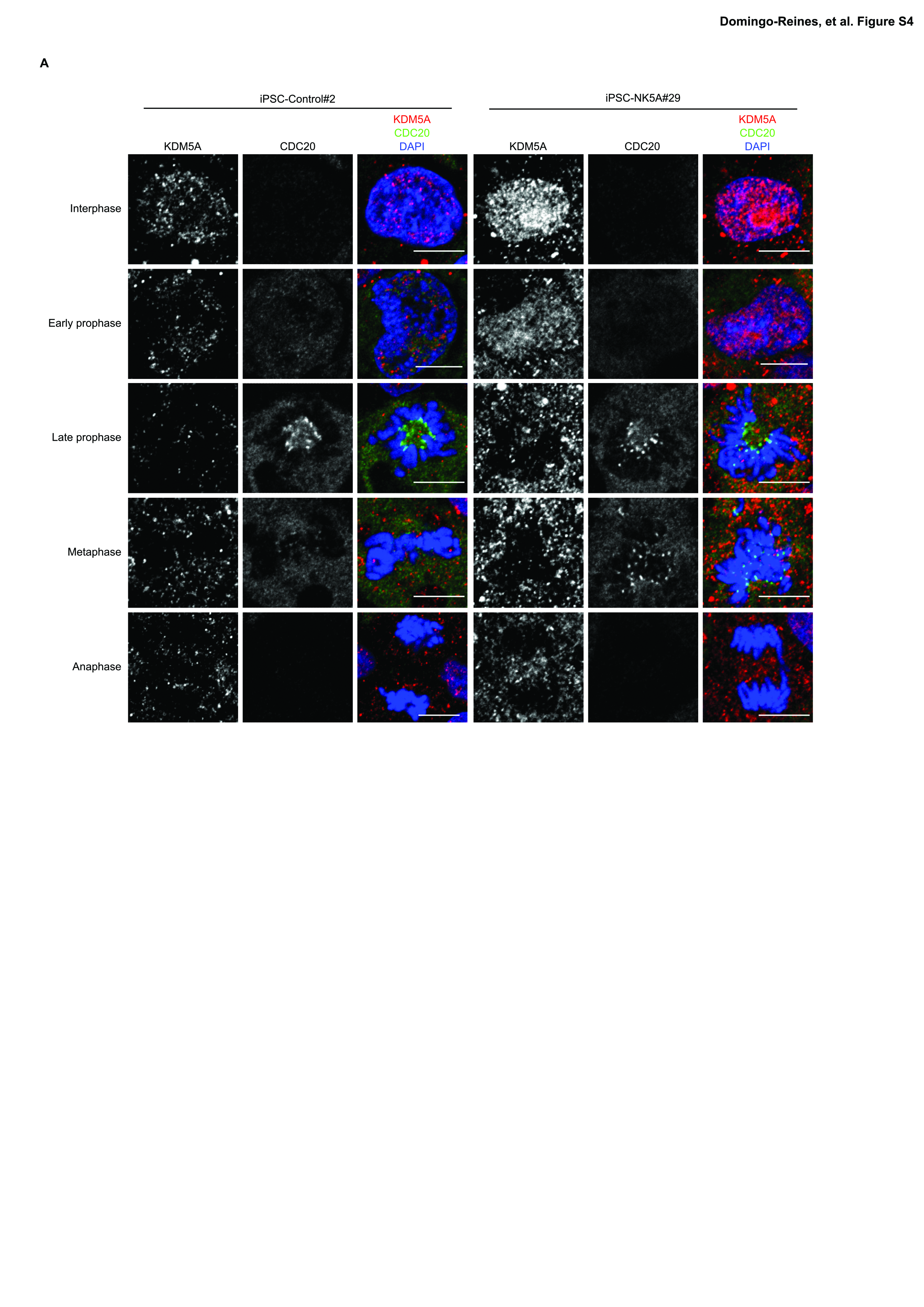

Supplement: Supplementary file 7 — Supplementary Figure 4 [file 41419_2023_5870_MOESM7_ESM.tif]

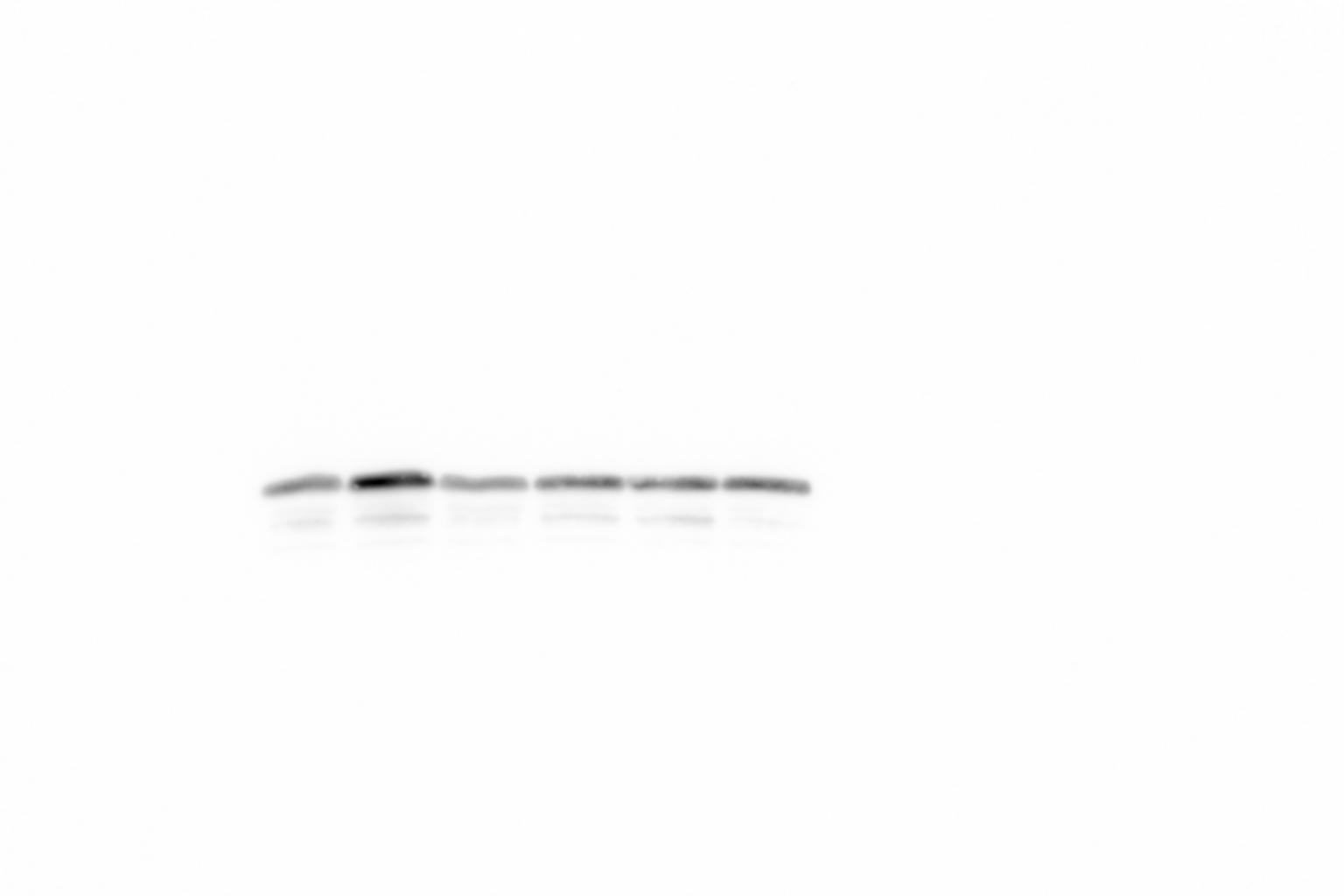

Supplement: Supplementary file 15 — Original WB H3 Figure 4E [file 41419_2023_5870_MOESM15_ESM.tif]

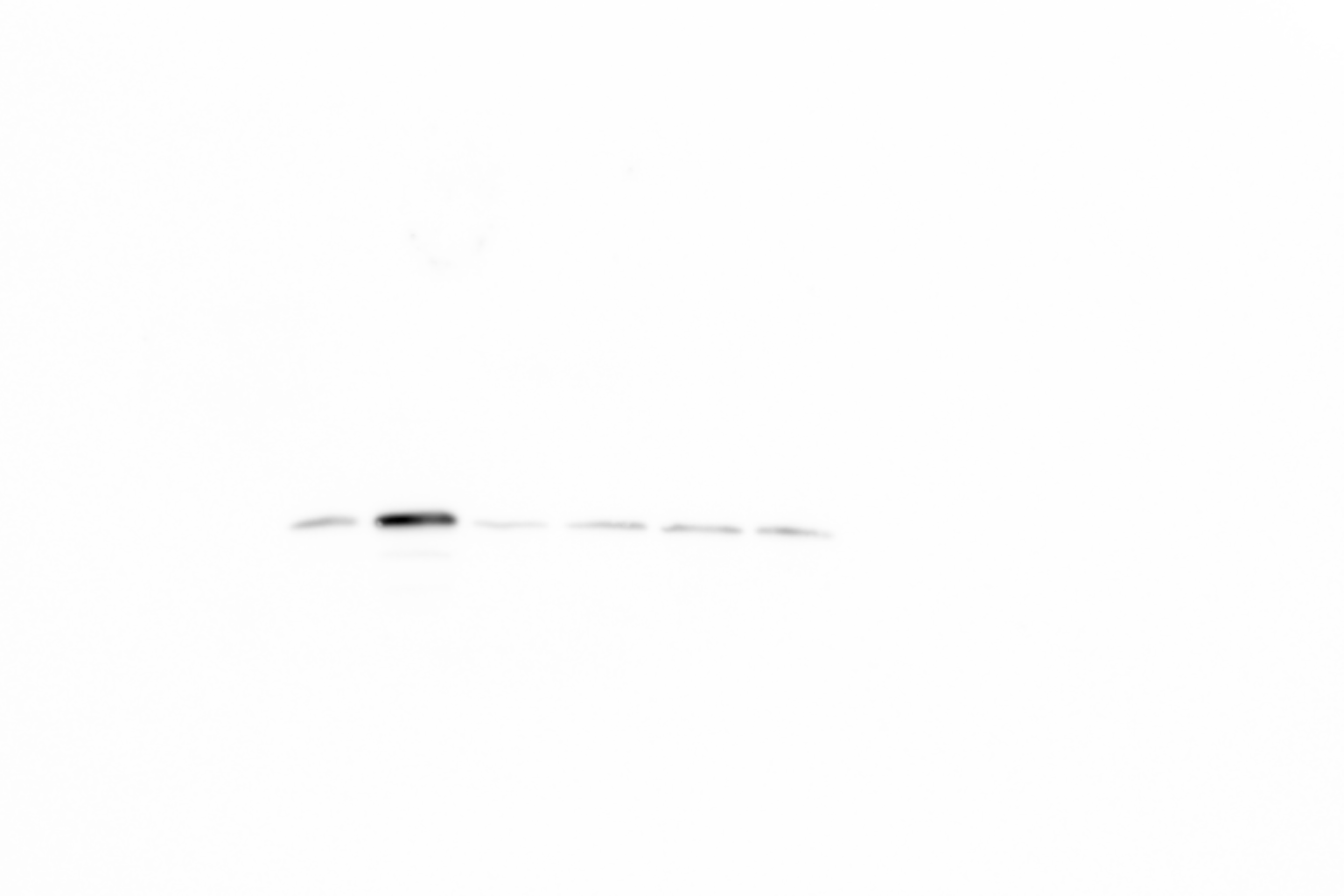

Supplement: Supplementary file 16 — Original WB H2AX Figure 4E [file 41419_2023_5870_MOESM16_ESM.tif]

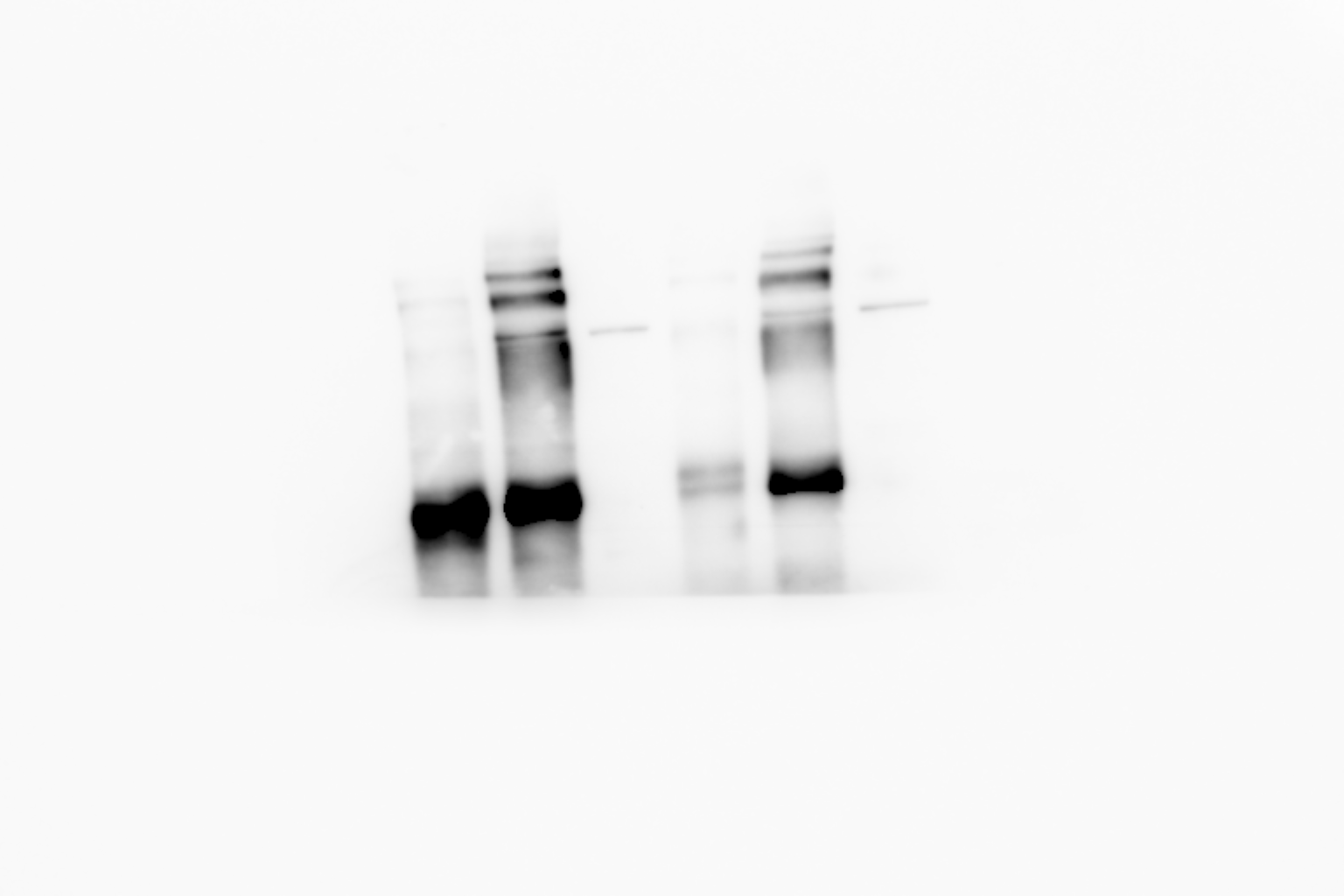

Supplement: Supplementary file 19 — Original WB KDM5A Figure 6F [file 41419_2023_5870_MOESM19_ESM.tif]

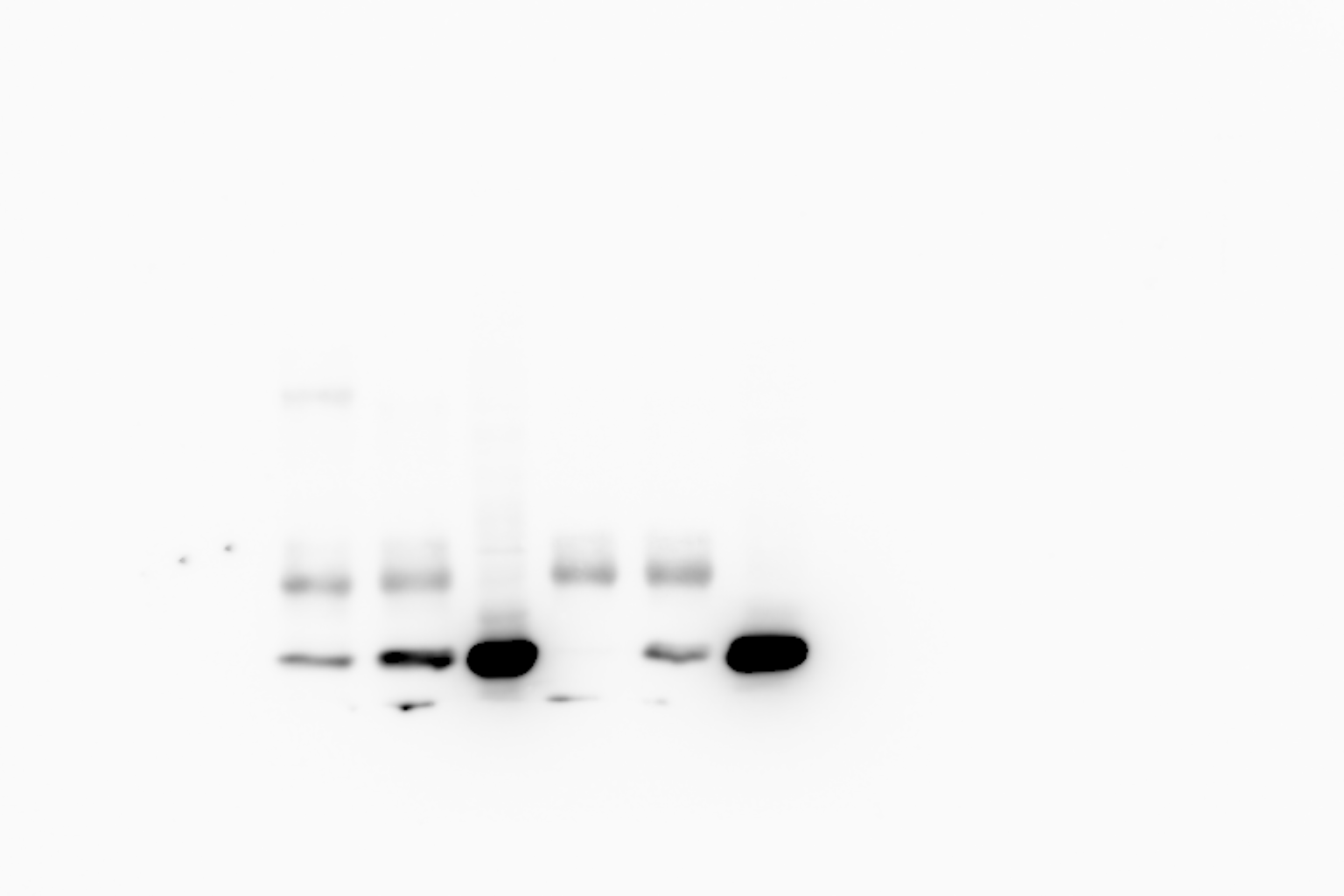

Supplement: Supplementary file 20 — Original WB RAE1 Figure 6F [file 41419_2023_5870_MOESM20_ESM.tif]
